# Pulmonary ventilation and gas exchange during prolonged exercise in humans: Influence of dehydration, hyperthermia and sympathoadrenal activity

**DOI:** 10.1113/EP090909

**Published:** 2023-01-09

**Authors:** José González‐Alonso, José A. L. Calbet, Ricardo Mora‐Rodríguez, Pascale Kippelen

**Affiliations:** ^1^ Division of Sport Health and Exercise Sciences Department of Life Sciences Brunel University London Uxbridge UK; ^2^ Department of Physical Education & Research Institute for Biomedical and Health Sciences (IUIBS) University of Las Palmas de Gran Canaria Gran Canaria Spain; ^3^ Department of Physical Performance Norwegian School of Sport Sciences Oslo Norway; ^4^ Department of Physical Activity and Sport Sciences University of Castilla‐La Mancha Toledo Spain

**Keywords:** blood gases, body fluids, temperature, ventilation

## Abstract

The mechanisms driving hyperthermic hyperventilation during exercise are unclear. In a series of retrospective analyses, we evaluated the impact of combined versus isolated dehydration and hyperthermia and the effects of sympathoadrenal discharge on ventilation and pulmonary gas exchange during prolonged intense exercise. In the first study, endurance‐trained males performed two submaximal cycling exercise trials in the heat. On day 1, participants cycled until volitional exhaustion (135 ± 11 min) while experiencing progressive dehydration and hyperthermia. On day 2, participants maintained euhydration and core temperature (*T*_c_) during a time‐matched exercise (control). At rest and during the first 20 min of exercise, pulmonary ventilation (${\dot{V}}_{E}$), arterial blood gases, CO_2_ output and O_2_ uptake were similar in both trials. At 135 ± 11 min, however, ${\dot{V}}_{E}$ was elevated with dehydration and hyperthermia, and this was accompanied by lower arterial partial pressure of CO_2_, higher breathing frequency, arterial partial pressure of O_2_, arteriovenous CO_2_ and O_2_ differences, and elevated CO_2_ output and unchanged O_2_ uptake despite a reduced pulmonary circulation. The increased ${\dot{V}}_{E}$ was closely related to the rise in *T*_c_ and circulating catecholamines (*R* ^2^ ≥ 0.818, *P* ≤ 0.034). In three additional studies in different participants, hyperthermia independently increased ${\dot{V}}_{E}$ to an extent similar to combined dehydration and hyperthermia, whereas prevention of hyperthermia in dehydrated individuals restored ${\dot{V}}_{E}$ to control levels. Furthermore, adrenaline infusion during exercise elevated both *T*_c_ and ${\dot{V}}_{E}$. These findings indicate that: (1) adjustments in pulmonary gas exchange limit homeostatic disturbances in the face of a blunted pulmonary circulation; (2) hyperthermia is the main stimulus increasing ventilation during prolonged intense exercise; and (3) sympathoadrenal activation might partly mediate the hyperthermic hyperventilation.

## Abstract (highlights)

- **What is the central question of the study?**
  Ventilation increases during prolonged intense exercise, but the impact of dehydration and hyperthermia, with associated blunting of pulmonary circulation, and independent influences of dehydration, hyperthermia and sympathoadrenal discharge on ventilatory and pulmonary gas exchange responses remain unclear.
- **What is the main finding and its importance?**
  Dehydration and hyperthermia led to hyperventilation and compensatory adjustments in pulmonary CO_2_ and O_2_ exchange, such that CO_2_ output increased and O_2_ uptake remained unchanged despite the blunted circulation. Isolated hyperthermia and adrenaline infusion, but not isolated dehydration, increased ventilation to levels similar to combined dehydration and hyperthermia. Hyperthermia is the main stimulus increasing ventilation during prolonged intense exercise, partly via sympathoadrenal activation.

## 1 INTRODUCTION

During prolonged intense exercise, pulmonary ventilation (${\dot{V}}_{E}$) increases out of proportion to the metabolic needs of active tissues and organs (Kearon et al., 1991). Evidence from numerous experimental studies has established that an elevation in core temperature is involved in the excessive rise in ${\dot{V}}_{E}$, particularly during exercise in uncompensable hot environments when marked internal hyperthermia is apparent (Hayashi et al., 2006; MacDougal et al., 1974; Nybo & Nielsen, 2001). Through an increase in alveolar ventilation and additional removal of CO_2_, hyperthermia‐induced hyperventilation challenges blood gas homeostasis, causing a state of hypocapnia and subsequent cerebral hypoperfusion (Nybo & Nielsen, 2001; Trangmar et al., 2015; Tsuji et al., 2015).

Fluid losses through thermoregulatory sweating often exceed fluid intake during prolonged intense exercise, leading to dehydration. The resulting hypovolaemia and hyperthermia are associated with reductions in peripheral and systemic blood flow (González‐Alonso et al., 1998, 1995, 1997; Montain & Coyle, 1992; Travers et al., 2020; Watanabe et al., 2020). Given that the pulmonary circulation reflects the systemic circulatory response, blunting of blood supply by way of hyperthermia and dehydration might interfere with pulmonary gas exchange and blood gases homeostasis. To date, no direct evaluation (via arterial blood sampling) of the combined effects of dehydration and hyperthermia on pulmonary ventilation and pulmonary gas exchange has been conducted during endurance exercise. A single human‐based study showed that hypohydration did not alter the ventilatory response to prolonged exercise in the heat, but no arterial blood gas measurements were available (Fujii et al., 2008b). Moreover, these results were obtained at moderate intensity [i.e., 50% of maximal oxygen uptake (${\dot{V}}_{O_{2}\max}$)], with similar core temperatures in the experimental (dehydration; ∼3.9% body mass loss) and control (euhydration) conditions. It is not known whether comparable findings would be obtained during more intense exercise (Kearon et al., 1991), when a similar level of dehydration is accompanied by marked internal body hyperthermia, cardiovascular strain and stronger ventilatory stimuli (such as elevations in plasma catecholamines, hyperosmolality, hypovolaemia and hyperkalaemia).

How pulmonary ventilation is controlled during exercise with hydration and thermal strain remains unresolved. When individuals become dehydrated during prolonged intense exercise, factors such as chemosensory discharge induced by increased blood osmolarity, detection of hyperosmolarity by brain osmoreceptors, hypovolaemia and/or increased release of ventilatory stimuli (Fujii et al., 2008b) could all modulate the breathing response. Likewise, the concomitant core hyperthermia could drive the rise in ventilation owing to high temperatures in the hypothalamus, medulla oblongata, spinal cord and/or skeletal muscles (Chai & Lin, 1972; Hertel et al., 1976; Holmes et al., 1960; Kumazawa & Mizumura, 1977; Tryba et al., 2003). However, the independent effects of dehydration‐induced hyperosmolality and hypovolemia and the separate influence of internal body hyperthermia on the ventilatory response to prolonged exercise have not been documented. Elucidation of the isolated effects of dehydration and hyperthermia enables identification of their respective contribution to the combined response.

Dehydration‐induced physiological strain during prolonged intense exercise is accompanied by significant increases in circulating catecholamines (González‐Alonso et al., 1995), which are likely to be coupled to a rise in sympathetic nerve activity to multiple organs and tissues, including skeletal muscles (Ray, 1993; Saito et al., 1997). Adrenaline has a well‐established thermogenic effect and, in resting humans, exogenous infusions of adrenaline and noradrenaline have been shown to increase ventilation (Butland et al., 1982; Heistad et al., 1972), with β‐adrenergic blockade attenuating the response (Heistad et al., 1972). It has not yet been established whether adrenaline can modulate the ventilatory response during exercise‐induced dehydration and hyperthermia in humans.

The aims of this investigation were threefold: (1) to characterize the impact of combined dehydration and hyperthermia and the associated blunting of pulmonary and systemic circulations on ventilation and pulmonary gas exchange during intense endurance exercise; (2) to isolate the contribution of dehydration and hyperthermia on the ventilatory response to exercise in the heat; and (3) to discern the influence of sympathoadrenal activity on ventilation during exercise‐induced dehydration and hyperthermia. Our hypotheses were as follows: (1) compensatory adjustments in pulmonary gas exchange will occur during prolonged intense exercise in the heat, such that arterial blood gas and acid–base disturbances are minimized; (2) hyperthermia, but not dehydration, will independently increase ventilation during prolonged intense exercise; and (3) adrenaline infusion during prolonged exercise in the heat will increase ventilation significantly. These hypotheses were tested in a series of human experiments, in which body fluid status, core temperature and circulating catecholamines were manipulated and ventilatory responses, pulmonary inspiratory and expiratory gases, arterial blood gases and cardiac output ($\dot{Q}$) were measured.

## 2 METHODS

### 2.1 Participants

Twenty‐nine endurance‐trained male cyclists took part in four studies. The participant demographics, reported in Table 1, were similar across the studies. The participants had a mean (±SD) age of 25 ± 5 years, body mass of 71.3 ± 6.4 kg, height of 178 ± 6 cm, maximal heart rate of 188 ± 6 beats min^−1^ and ${\dot{V}}_{O_{2}\max}$ of 4.5 ± 0.4 L min^−1^ or 63 ± 5 ml kg^−1^ min^−1^.

**TABLE 1:** Demographics of participants

**Variable**	**Combined dehydration and hyperthermia (study 1)**	**Combined dehydration and hyperthermia (study 2)**	**Isolated hyperthermia (study 2)**	**Isolated dehydration (study 3)**	**Hyperthermia and adrenaline infusion (study 4)**
Age, years	27 ± 2	25 ± 4	25 ± 4	25 ± 4	27 ± 7
Height, cm	184 ± 7	178 ± 6	179 ± 7	179 ± 7	176 ± 5
Body mass, kg	78.5 ± 7.2	71.7 ± 7.1	70.9 ± 3.4	70.9 ± 3.4	69.9 ± 3.8
${\dot{V}}_{O_{2}\max}$, L min^−1^	4.9 ± 0.6	4.5 ± 0.4	4.4 ± 0.4	4.4 ± 0.4	4.4 ± 0.4
${\dot{V}}_{O_{2}\max}$, ml kg^−1^ min^−1^	63 ± 7	63 ± 5	62 ± 4	62 ± 4	64 ± 5
Maximal heart rate, beats min^−1^	197 ± 11	188 ± 6	186 ± 6	186 ± 6	187 ± 6

*Note*: Data are means ± SD for 7, 7, 8 and 7 male participants for study 1, 2, 3 and 4, respectively. The effects of combined dehydration and hyperthermia during the 30 min intense exercise bouts (study 2) include the data of 15 additional participants from two published studies with the same participant demographics and experimental design (total *n* = 22) (González‐Alonso, Mora‐Rodríguez et al., 1999; González‐Alonso et al., 2000).

Abbreviation: ${\dot{V}}_{O_{2}\max}$, maximal pulmonary oxygen uptake.

### 2.2 Ethical approval

All participants were fully informed of any risks and discomforts associated with the experiments before giving their informed written consent to participate. The studies conformed to the standards set by the *Declaration of Helsinki*, except for registration in a database (studies 2–4). Studies were approved by the Ethics Committee of Copenhagen and Frederiksberg communities, Denmark (study 1; KF‐01‐045/96) and the Internal Review Board at The University of Texas at Austin, USA (studies 2–4). All individuals had participated in experiments involving endurance performance in hot environments before undergoing these experiments. Furthermore, participants acclimated to the heat and adapted to large volumes of fluid ingestion before the experimental trials by performing four practice trials (2 h cycling exercise at ∼60% ${\dot{V}}_{O_{2}\max}$ in a 35°C environment). Reports of the cardiovascular, metabolic and thermal responses to exercise with varied hydration status from these studies have been previously published (González‐Alonso et al., 1998, 1997, 2000; González‐Alonso, Calbet et al., 1999; González‐Alonso, Mora‐Rodríguez et al., 1999; Mora‐Rodríguez et al., 1996). The present manuscript focuses on new ventilatory and pulmonary gas exchange data and analyses. The initial findings of this investigation were presented at the annual conference Physiology 2021.

### 2.3 Experimental protocols

In the four studies, the experimental protocols had two separate experimental days, separated by 3–7 days, with trials ran in randomized order and counterbalanced across participants (except study 1, in which the dehydration with hyperthermia trial always preceded the control trial). On the day before experimental testing, the hydration status of the participants was standardized by having them adopt the same diet, exercise (1 h of low‐intensity cycling) and fluid intake regimen. The participants reported to the laboratory at the same time of day, ∼1−4 h before the experiment, after the ingestion of a plentiful breakfast and 200−300 ml of fluid. The oral fluid‐replacement solution in all studies was made from a commercially available sports drink. The temperature of the solution during exercise was ∼38°C to minimize fluctuations in core temperature.

#### 2.3.1 Study 1: Impact of hydration status on the ventilatory and pulmonary gas exchange responses to prolonged exercise in the heat

On two occasions, participants exercised continuously on a cycle ergometer (Monark 829E) in the heat (35°C, 41−46% relative humidity, 1−2 m s^−1^ fan speed) at a power output of 209 ± 28 W (mean ± SD) and pedalling frequency of 80−90 r.p.m., which elicited 61 ± 2% ${\dot{V}}_{O_{2}\max}$ early in exercise. In the first trial (dehydration and hyperthermia), they cycled until volitional exhaustion (135 ± 11 min) while developing significant dehydration and hyperthermia [3.9 ± 0.7% body mass loss, 39.7 ± 0.6°C core temperature (*T*_c_)]. During that trial, participants received only ∼0.8 L of fluid, with a total of 0.2 L of a concentrated carbohydrate–electrolyte solution ingested in three equal boluses at 30, 60 and 90 min of exercise and ∼0.6 L of saline solution infused i.v. at 20, 60, 90, 120 and 135 ± 11 min. Participants therefore replaced 28% of the sweat losses and lost 3.07 ± 0.74 kg. In the second trial (control), participants cycled for the same period of time, while maintaining euhydration by taking in fluid and stabilizing *T*_c_ at 38.2°C after 30 min of exercise. Participants received ∼4.3 L of fluid as 3.7 ± 0.3 L of diluted carbohydrate–electrolyte solution by mouth and ∼0.6 L of i.v. infused saline. The carbohydrate–electrolyte solution was ingested in equal boluses at 20, 35, 50, 65, 80, 95 and 110 min of exercise. This resulted in the complete maintenance of body mass.

Upon arrival in the laboratory, the participants rested in the supine position while two catheters were inserted under local anaesthesia using the Seldinger technique and sutured to the skin under local anesthesia: one in the femoral artery and another in the femoral vein of the right leg. Both catheters were inserted 1−2 cm below the inguinal ligament. After ∼1 h of rest in the supine position, participants walked to another room, were weighed naked and sat for 15 min in armchair while 10 ml blood samples were obtained simultaneously from the femoral artery and vein for later determination of baseline blood variables. Participants then entered a controlled‐environment chamber (35°C, 41−46% relative humidity) and sat on a bicycle ergometer while resting cardiopulmonary measurements were obtained. They then started to exercise.

During exercise, *T*_c_ and skin temperature (*T*_sk_) were recorded continuously, whereas the ventilatory responses, oxygen uptake (${\dot{V}}_{O_{2}}$) and carbon dioxide output (${\dot{V}}_{CO_{2}}$), were measured over a 5 min period beginning at 5, 40, 80, 105 and 125−135 min of exercise and during each determination of cardiac output. Cardiac output was measured in triplicate over a 10 min period after the ${\dot{V}}_{O_{2}}$ and ${\dot{V}}_{CO_{2}}$ measurements. Upon completion of the exercise, catheters were removed and postexercise naked body mass was recorded.

#### 2.3.2 Study 2: Partitioning the effects of hyperthermia and dehydration on the ventilatory response to prolonged exercise—Effects of isolated hyperthermia

On two occasions, participants completed a series of submaximal exercise bouts on a cycle ergometer (Monark 829E) that induced a state of dehydration and hyperthermia, isolated hyperthermia or euhydration (control). Before exercise, a resting baseline 10 ml forearm venous blood sample was obtained. Participants then cycled for 100 min at ∼60% of ${\dot{V}}_{O_{2}\max}$ (∼200 W; ∼80–90 r.p.m.) in the heat (35°C, 50% relative humidity, 1.5 m s^−1^ wind speed), ingesting different volumes of fluid (i.e., 0.2 ± 0.3 vs. 3.1 ± 0.8 L) and either became dehydrated or remained euhydrated. After the 100 min bout with full fluid replacement, participants rested for 45 min in a 23°C environment while drinking 0.3 ± 0.3 L of fluid. They then performed an additional 30 min bout of exercise in the heat (35.5°C, 53% relative humidity, 2 m s^−1^ wind speed) at ∼72 ± 2% of ${\dot{V}}_{O_{2}\max}$ (239 ± 23 W), which produced dehydration and hyperthermia (4.4 ± 0.2% body mass loss, *T*_c_ = 39.3 ± 0.3°C) while ventilatory responses were evaluated. In the alternative condition (euhydration), after the initial 100 min bout of exercise, participants rested for 15 min in the heat and drank 1.0 ± 0.3 L of warm fluid (38°C). During this period, they were partly covered to prevent core temperature from fully declining, while care was taken to prevent elevations in *T*_sk_, which averaged 35.2°C. They subsequently exercised for 30 min in the heat at ∼72 ± 2% of ${\dot{V}}_{O_{2}\max}$ while euhydrated but hyperthermic (isolated hyperthermia condition), with a *T*_c_ of 39.3 ± 0.3°C. To increase heat storage, for the first 10 min, participants exercised without fan cooling. For the period from 10 to 30 min, the fan speed was the same as in the dehydration and hyperthermia trial (i.e., 2 m s^−1^), resulting in similar *T*_c_ and *T*_sk_ during this time period in both trials. After completion of this first 30 min exercise bout, participants rested for 45 min in a 23°C environment to lower core temperature fully, while drinking 0.9 ± 0.3 L of fluid (22°C). They then performed a second 30 min bout of exercise in the heat while maintaining a low core temperature (*T*_c_ = 38.3 ± 0.3°C) when euhydrated (control conditions).

#### 2.3.3 Study 3: Establishing the effects of isolated dehydration on the ventilatory response to prolonged exercise

On two occasions, participants exercised at submaximal intensities on a cycle ergometer (Monark 829E) in a state of dehydration, rehydration or euhydration, while hyperthermia was prevented. Participants first cycled for 120 min at ∼60% of ${\dot{V}}_{O_{2}\max}$ (∼208 W) in the heat (35°C, 50% relative humidity, 1.5 m s^−1^ wind speed). By ingesting different volumes of fluid (3.2 ± 0.5 vs. 0.2 ± 0.2 L), they remained either euhydrated (control trial) or became dehydrated (i.e., 4.1 ± 0.3% body mass loss). Participants then rested for 45 min in a 23°C environment while drinking 0.6 ± 0.3 and 0.3 ± 0.3 L of fluid during the control and dehydration trials, respectively. Thereafter, ventilatory responses were evaluated as they performed two additional 30 min bouts of exercise (with 45 min of rest in between) at a constant work rate of 242 ± 24 W (70 ± 2% of ${\dot{V}}_{O_{2}\max}$) in a cold environment (2°C, with fans blowing to produce a windchill index of about −5°C). In the dehydration trial, cardiopulmonary responses in a dehydrated state were determined during the first 30 min bout of exercise. During the follow‐up rest period, participants were infused i.v. with 349 ± 60 ml of a blood volume expander (Macrodex; 6% w/v Dextran 70 in normal saline; Pharmacia Laboratories). This was preceded by infusion of 20 ml of Dextran 1 (Promit) to reduce the risk of anaphylactic reactions. In this trial (i.e., dehydration with blood volume restoration), blood volume was restored to control levels experienced during exercise while euhydrated, but the intracellular and interstitial bodily fluid compartments remained dehydrated (4.2 ± 0.1% body mass loss).

In studies 2 and 3, on their arrival in the laboratory, nude body mass was recorded, and participants were clothed in shorts, socks and cycling shoes. They then sat in the heat (35°C) for ≥20 min while an oesophageal thermistor was inserted, a Teflon catheter was inserted into an antecubital vein, and a baseline blood sample was obtained. On completion of the first 100−120 min of exercise during all trials (except the hyperthermia trial), participants removed their clothing, towelled dry, and their postexercise body mass was recorded. Skin thermistors were attached before each 30 min exercise bout, allowing continuous recording of *T*_c_ and *T*_sk_ during exercise. Measurements of the ventilatory responses, end‐tidal partial pressure of carbon dioxide ($P_{\mathrm{ETC}O_{2}}$), ${\dot{V}}_{O_{2}}$ and ${\dot{V}}_{CO_{2}}$ were obtained from 20 to 28 min of exercise, representing the steady‐state responses in each 30 min experimental bout. A 10 ml forearm venous blood sample was obtained at 30 min of exercise while participants were still pedalling.

#### 2.3.4 Study 4: Impact of adrenaline infusion on the ventilatory response to prolonged exercise in the heat

On two different occasions, participants exercised for 120 min on a cycle ergometer (Jaeger ERGOTEST) in a warm environment (33.1 ± 0.7°C with 1–2 m s^−1^ fan cooling) at a constant workload that elicited 65% of ${\dot{V}}_{O_{2}\max}$ initially (i.e., 216 ± 16 W; 80–90 r.p.m.). Upon arrival in the laboratory, two Teflon catheters were inserted into the antecubital veins of both arms of the participants for infusion and blood sampling. After 15 min of sitting on the ergometer, a resting blood sample was drawn and exercise began. From 30 to 120 min of exercise, saline or adrenaline solutions were continuously infused i.v. The infusion rates were controlled by a peristaltic pump (for saline) and a syringe pump (for adrenaline; Harvard Apparatus) to achieve the target adrenaline infusion rate of 0.1 μg kg^−1^ min^−1^. Pilot work indicated that this rate would increase the plasma adrenaline concentration to levels typically experienced during high‐intensity exercise (González‐Alonso & Calbet, 2003; Kreisman et al., 2000; Munch et al., 2014; Rosenmeier et al., 2004). The ${\dot{V}}_{E}$, ${\dot{V}}_{O_{2}}$, ${\dot{V}}_{CO_{2}}$ and breathing frequency (*f*_b_) were measured during a 5 min period beginning at 4, 40, 80 and 110 min of exercise. Arterial partial pressure of carbon dioxide ($P_{\mathrm{aC}O_{2}}$) was estimated in quadruplicate from $P_{\mathrm{ETC}O_{2}}$ measurements obtained after the ${\dot{V}}_{O_{2}}$ determinations. Blood samples (15 ml) were obtained at 30 min intervals during exercise. When the exercise period was completed, infusion was stopped, and participants continued pedalling at a reduced work rate (∼150 W) for an additional 10 min to facilitate ‘washout’ of the infused adrenaline. During the entire adrenaline trial, the ECG was monitored, and it was observed that normal sinus rhythm was maintained in all participants. A total of 0.8 ± 0.1 L of fluid was infused i.v. in the saline and adrenaline infusion trials, such that 30 ± 7% of sweat losses during exercise were replaced.

### 2.4 Analytical methods

#### 2.4.1 Pulmonary ventilation, oxygen consumption, carbon dioxide production and cardiac output

Ventilatory parameters [${\dot{V}}_{E}$, *f*_b_ and tidal volume (*V*_T_)] and pulmonary ${\dot{V}}_{O_{2}}$ and ${\dot{V}}_{CO_{2}}$ were measured online with either a Medgraphics CPX/D metabolic cart (Saint Paul, Minneapolis, MN, USA; in study 1) or a custom‐made metabolic cart (studies 2–4) in which participants breathed through a Daniel's valve connected to a mixing chamber on the expirate side and to a dry gas meter (CD4; Parkinson‐Cowan) on the inspirate side. Expired air was analysed for O_2_ (S‐3A/I; Ametek) and CO_2_ (CD‐3A; Ametek) concentrations. Both analysers and the gas meter were interfaced with a laboratory computer (Apple IIe) through an analog‐to‐digital conversion board (REP‐200B; Rayfield, Chicago, IL, USA).

Cardiac output was determined using the computerized version of the CO_2_ rebreathing technique of Collier (1956) and adjusted for arterial haemoglobin concentration ([Hb]) (McHardy, 1967), $P_{\mathrm{aC}O_{2}}$ and arterial oxygen saturation ($S_{aO_{2}}$). Cardiac output was calculated by using the Fick equation for CO_2_ [cardiac output = ${\dot{V}}_{CO_{2}}$ divided by mixed‐venous CO_2_ content ($C_{\bar{v}CO_{2}}$) minus arterial CO_2_ content ($C_{\mathrm{aC}O_{2}}$)]. The CO_2_ contents were determined by estimating the respective partial pressures, then converting the partial pressures into contents using a standard CO_2_ dissociation curve (Comroe et al., 1962). Expired air was sampled from a mixing chamber and analysed for O_2_ and CO_2_ concentration as described above. The $P_{\mathrm{ETC}O_{2}}$ was determined on a breath‐by‐breath basis by continuous sampling at the mouthpiece by using a CO_2_ analyser (CD‐3A; Ametek) interfaced with a laboratory computer. The mixed‐venous partial pressure of CO_2_ ($P_{\bar{v}CO_{2}}$) was estimated from the $P_{CO_{2}}$ equilibrium attained during the rebreathing procedure. The criteria for CO_2_‐rebreathing equilibrium were that equilibrium was obtained within the 15 s of rebreathing procedure and that the steady‐state $P_{CO_{2}}$ varied by <1 mmHg over a 5 s period.

#### 2.4.2 Blood analysis

Haematocrit was measured in triplicate after microcentrifugation. In study 1, [Hb] and $S_{aO_{2}}$ were determined spectrophotometrically (OSM‐2 Hemoximeter; Radiometer, Copenhagen, Denmark). In studies 2–4, [Hb] was measured using the cyanmethaemoglobin technique. The $P_{aO_{2}}$, femoral venous partial pressure of O_2_ ($P_{\mathrm{fv}O_{2}}$) $P_{CO_{2}}$ and pH were determined using the Astrup technique (ABL30; Radiometer) and corrected for measured blood temperature, whereas [HCO_3_^−^] was calculated using the Siggaard–Andersen equation (1974). Actual base excess (ABE) refers to the difference between the observed and normal buffer base concentration or the amount of acid or base needed to return the pH to 7.4 in the setting of a normal $P_{\mathrm{aC}O_{2}}$. A negative ABE indicates reduced base (acidosis), whereas a positive value indicates excess base (alkalosis). Serum osmolality was determined using the freezing point depression technique (3MO; Advanced Instruments). For catecholamine determination, blood samples (3 ml) were placed promptly into prechilled tubes containing 4.5 mg of reduced glutathione, 50 IU of sodium heparin and 20 μl of 0.24 M EGTA. Blood was subsequently centrifuged at 4°C and plasma separated and stored frozen at −80°C until analysis. Plasma catecholamine concentrations were measured in duplicate using a radioenzymatic assay (Christiansen et al., 1980) or high‐performance liquid chromatography with electrochemical detection (Hjemdahl, 1984).

### 2.5 Temperature

The *T*_c_ was measured with a thermistor (Ellab or YSI 491) inserted through the nasal passage to a distance equal to one‐quarter of the standing height of the participant. Mean skin temperature was calculated from six sites (i.e., upper arm, forearm, chest, back, thigh and calf) using the weighting method of Hardy and DuBois (1938). Skin thermistors (Ellab or YSI 409A) were interfaced with a telethermometer (YSI 2100) or temperature recorder (CTF 90008 precision thermometer; Ellab) interfaced to an IBM‐AT computer. Blood temperature in study 1 was recorded using a data‐acquisition system (MacLab 8s; ADInstruments, Sydney, NSW, Australia) interfaced to a computer (Macintosh Performa).

### 2.6 Calculations

Arterial and femoral venous whole‐blood CO_2_ content ($C_{\mathrm{fvC}O_{2}}$) was determined from measured blood [Hb], temperature, $S_{aO_{2}}$, pH and $P_{CO_{2}}$ according to the model proposed by Douglas et al. (1988), or estimated from the $P_{\mathrm{ETC}O_{2}}$ as described above (Jones et al., 1979). The $C_{\bar{v}CO_{2}}$ was estimated from the $P_{\bar{v}CO_{2}}$ obtained during the CO_2_ rebreathing measurements, as reported above. Alveolar ventilation (${\dot{V}}_{A}$) was assessed from the values of $P_{\mathrm{ETC}O_{2}}$ [used as a surrogate of alveolar CO_2_ pressure ($P_{\mathrm{AC}O_{2}}$)] and ${\dot{V}}_{CO_{2}}$: ${\dot{V}}_{A}$ = 0.863 × ${\dot{V}}_{CO_{2}}$/$P_{\mathrm{AC}O_{2}}$ [0.863 being the constant to adapt standard temperature and pressure dry (STPD) to body temperature, ambient pressure, saturated (BTPS)]. In one participant, ${\dot{V}}_{A}$ was not calculated because his respiratory exchange ratio went above 1.0 towards the end of one of the exercise trials. In all the other participants, anatomical dead space (*V*_D_) was calculated from total and alveolar ventilations: *V*_D_ = (${\dot{V}}_{E}$ − ${\dot{V}}_{A}$)/*f*_b_.

Blood volume values were calculated by predicting the absolute resting euhydrated values (Sawka et al., 1992), then calculating the changes in blood volume from the changes in [Hb] (Dill & Costill, 1974). Percentage dehydration was estimated from the difference in body mass between pre‐ and postexercise, with a correction factor applied to account for exchanges of O_2_ and CO_2_ (Nadel et al., 1979). Nude body mass was determined on a platform scale (FW 150 KAI; Acme Scale) with an accuracy of ±20 g.

### 2.7 Statistical analysis

Data from a given study were analysed by using a one‐way (condition) or two‐way (trial‐by‐time) ANOVA with repeated measures. Mauchley's test was used to test the assumption of sphericity. In cases where this assumption was violated, a Greenhouse–Geisser correction factor was applied. After a significant *F*‐test, pairwise differences were identified by using the Bonferroni post hoc procedure. Regression analysis was used to establish the relationships between ventilatory parameters and core temperature, $P_{\mathrm{aC}O_{2}}$ and catecholamines. All statistical analyses were conducted using MAC SPSS Statistics (v.26; IBM, Armonk, NY, USA). Data are presented as the means ± SD. The level of significance was set at *P* < 0.05.

## 3 RESULTS

### 3.1 Study 1: Impact of hydration status on the ventilatory and pulmonary gas exchange responses to prolonged exercise in the heat

**FIGURE 1:**
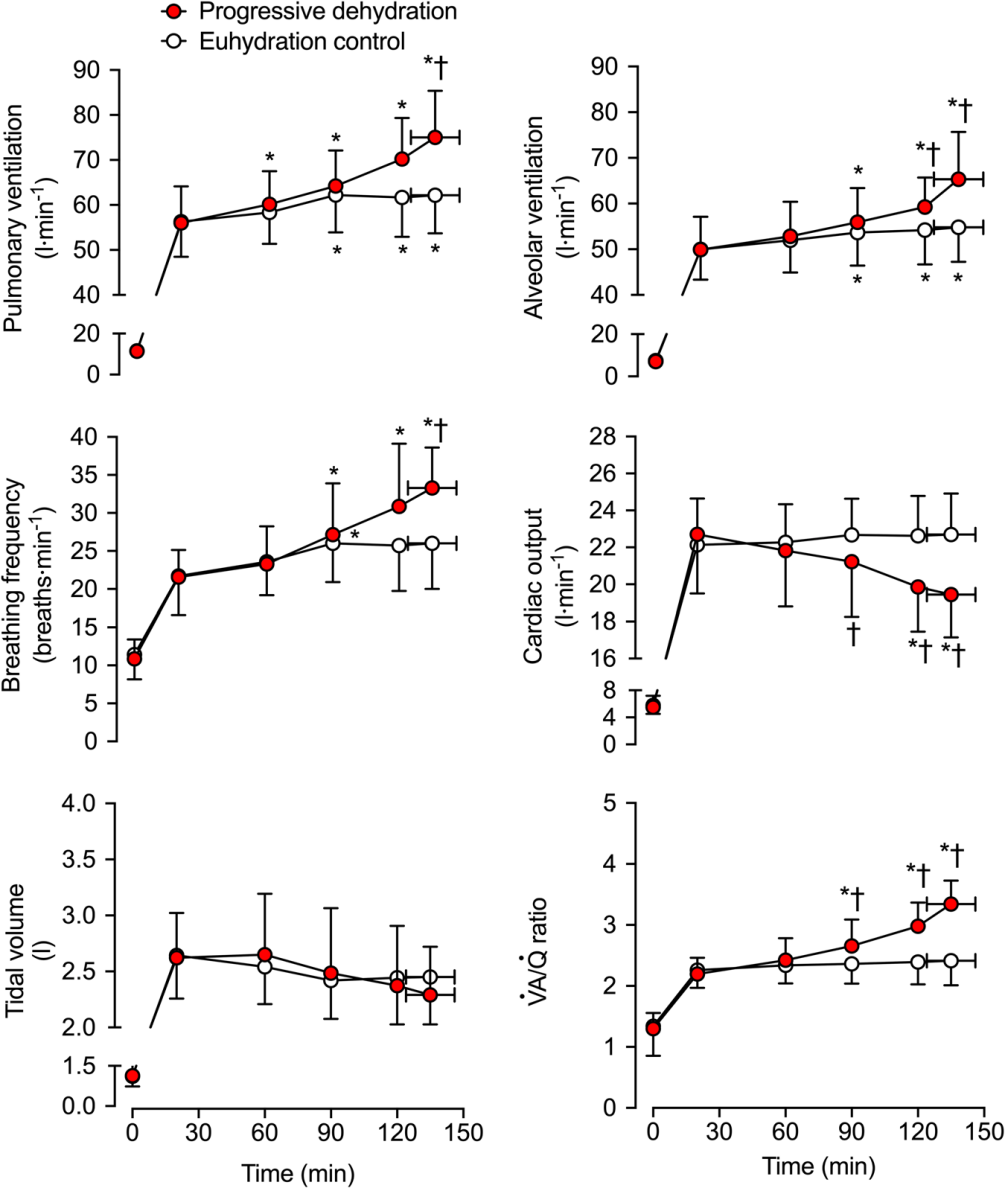
Ventilatory, respiratory and perfusion responses during prolonged intense exercise with varied hydration status. Pulmonary ventilation, breathing frequency, tidal volume, alveolar ventilation (${\dot{V}}_{A}$), cardiac output ($\dot{Q}$) and ${\dot{V}}_{A}$/$\dot{Q}$ ratio during the progressive dehydration and euhydration control trials. Data are shown as the mean ± SD for seven subjects, except for ${\dot{V}}_{A}$ and ${\dot{V}}_{A}$/$\dot{Q}$, where data are from six subjects. ^*^Significantly different from 20 min, *P <* 0.05. ^†^Significantly different from euhydration control, *P <* 0.05

Hydration indexes, body temperature, ventilatory responses and blood parameters were all similar at rest and after 20–30 min of exercise in both trials (all *P >* 0.05; Table 2; Figure 1). During the combined dehydration and hyperthermia trial, the participants lost 3.9 ± 0.7% of their body mass whilst *T*_c_ increased progressively to 39.7 ± 0.6°C. In contrast, euhydration was maintained and *T*_c_ stabilized at 38.2 ± 0.3°C in the control trial (Table 2). Reflecting differences in hydration status after the first hour of exercise, arterial haemoglobin concentration ([Hb]_a_; *P* ≤ 0.031) and arterial osmolality (*P* ≤ 0.004) were elevated, whereas estimated blood volume (*P <* 0.030) was reduced in the dehydration with hyperthermia trial compared with the euhydration trial (Table 2; Figure 1).

**TABLE 2:** Thermal and arterial blood variables (including plasma catecholamines) and end‐tidal gases during prolonged intense exercise with progressive dehydration compared with the euhydration control trial

		**Time (min)**					
**Variable**	**Trial**	**0**	**20**	**60**	**90**	**120**	**134 ± 11**
*T*_c_, °C	Dehydration	37.1 ± 0.4	38.1 ± 0.3	38.5 ± 0.4	38.9 ± 0.5^†^	39.4 ± 0.5^*^, ^†^	39.7 ± 0.6^*^, ^†^
	Euhydration	37.0 ± 0.2	37.9 ± 0.2	38.1 ± 0.2	38.2 ± 0.2	38.2 ± 0.3	38.3 ± 0.3
*T*_sk_, °C	Dehydration	35.1 ± 0.6	34.6 ± 0.5	34.8 ± 0.4	35.0 ± 0.3	35.4 ± 0.7	35.4 ± 0.7
	Euhydration	34.7 ± 0.7	34.0 ± 0.8	34.1 ± 0.7	34.3 ± 0.7	34.6 ± 0.7	34.6 ± 0.8
Blood volume, L	Dehydration	5.22 ± 0.35	5.05 ± 0.36	4.99 ± 0.38^†^	4.94 ± 0.37^†^	4.87 ± 0.36^†^	4.85 ± 0.35^*^, ^†^
	Euhydration	5.20 ± 0.32	5.08 ± 0.37	5.15 ± 0.35	5.27 ± 0.36	5.14 ± 0.38	5.14 ± 0.36
Osmolality_a_, mosmol kg^−1^	Dehydration	288 ± 3	295 ± 3	299 ± 3*^†^	302 ± 3*^†^	305 ± 3^*^, ^†^	308 ± 5^*^, ^†^
	Euhydration	287 ± 5	292 ± 3	290 ± 8	289 ± 8	287 ± 8	287 ± 2*
$P_{\mathrm{ETC}O_{2}}$, mmHg	Dehydration	42 ± 4	48 ± 4	45 ± 4*	43 ± 3*	38 ± 6^*^, ^†^	37 ± 5^*^, ^†^
	Euhydration	41 ± 3	48 ± 4	46 ± 5*	44 ± 5*	44 ± 4*	43 ± 5*
$P_{\bar{v}{\mathrm{CO}}_{2}}$, mmHg	Dehydration	60 ± 3	74 ± 4	73 ± 3	71 ± 3	69 ± 4*	68 ± 4*
	Euhydration	60 ± 4	75 ± 3	74 ± 4	73 ± 4	72 ± 4*	72 ± 4*
$P_{\mathrm{ET}O_{2}}$, mmHg	Dehydration	109 ± 5	104 ± 6	107 ± 7*	109 ± 6*	114 ± 10*	116 ± 9^*^, ^†^
	Euhydration	109 ± 3	103 ± 6	104 ± 5	107 ± 5	106 ± 5	106 ± 5
[NA]_a_, nmol L^−1^	Dehydration	2.3 ± 0.6	9.0 ± 2.6	11.8 ± 3.5^*^, ^†^	14.6 ± 3.7^*^, ^†^	17.0 ± 3.8^*^, ^†^	18.5 ± 3.0^*^, ^†^
	Euhydration	2.3 ± 0.4	8.5 ± 2.1	9.2 ± 2.4	10.8 ± 3.4	11.7 ± 3.8	11.3 ± 4.4
[A]_a_, nmol L^−1^	Dehydration	1.0 ± 0.3	1.3 ± 0.3	1.6 ± 0.4	1.8 ± 0.5^†^	1.9 ± 0.6^*^, ^†^	2.1 ± 0.6^*^, ^†^
	Euhydration	1.0 ± 0.3	1.3 ± 0.3	1.3 ± 0.4	1.4 ± 0.4	1.3 ± 0.3	1.5 ± 0.2

*Note*: Values are means ± SD for seven participants in study 1.

Abbreviaions: [A]_a_, arterial adrenaline concentration; Blood volume, estimated blood volume; [HCO_3_^−^]_a_, bicarbonate concentration; [NA]_a_, arterial noradrenaline concentration; Osmolality_a_, arterial osmolality; $P_{\mathrm{ETC}O_{2}}$, end‐tidal partial pressure of CO_2_; $P_{\mathrm{ET}O_{2}}$, end‐tidal partial pressure of O_2_; pH_a_, arterial pH; $P_{\bar{v}{\mathrm{CO}}_{2}}$, mixed‐venous partial pressure of CO_2_; *T*_c_, core temperature; *T*_sk_, mean skin temperature.

^*^ Significantly different from 20 min, *P <* 0.05.

^†^ Significantly different from euhydration control trial, *P <* 0.05.

**FIGURE 2:**
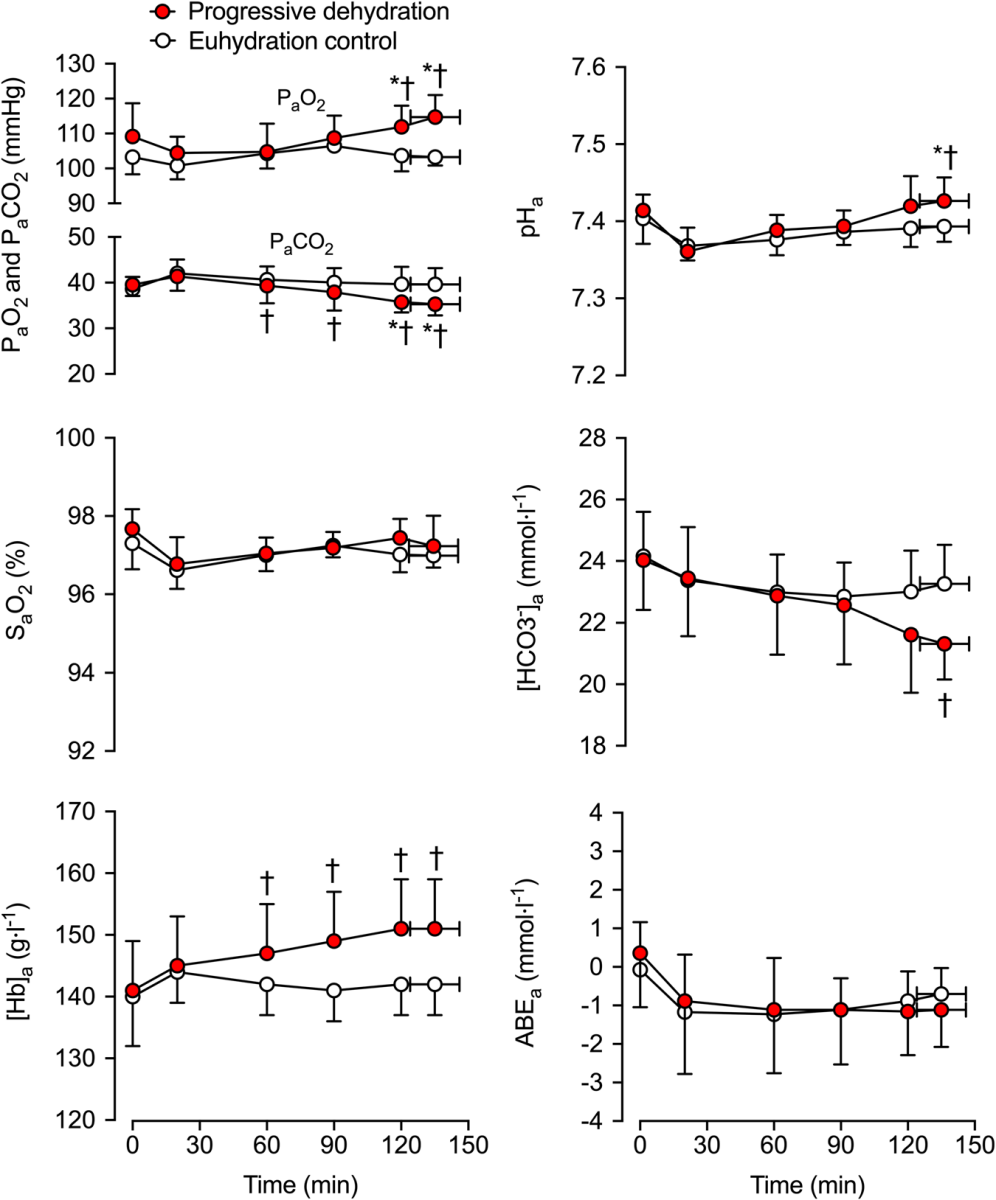
Arterial blood gases and acid–base balance during prolonged intense exercise with varied hydration status. The arterial partial pressure of carbon dioxide ($P_{\mathrm{aC}O_{2}}$), arterial partial pressure of oxygen ($P_{aO_{2}}$), arterial oxygen saturation ($S_{aO_{2}}$), arterial CO_2_ content ($C_{\mathrm{aC}O_{2}}$), arterial O_2_ content ($C_{aO_{2}}$), arterial pH (pH_a_), arterial bicarbonate concentration ([HCO_3_^−^]_a_) and arterial actual base excess (ABE_a_) were measured during the progressive dehydration and euhydration control trials. Data are shown as the mean ± SD for seven subjects. ^*^Significantly different from 20 min, *P <* 0.05. ^†^Significantly different from euhydration control, *P <* 0.05

**FIGURE 3:**
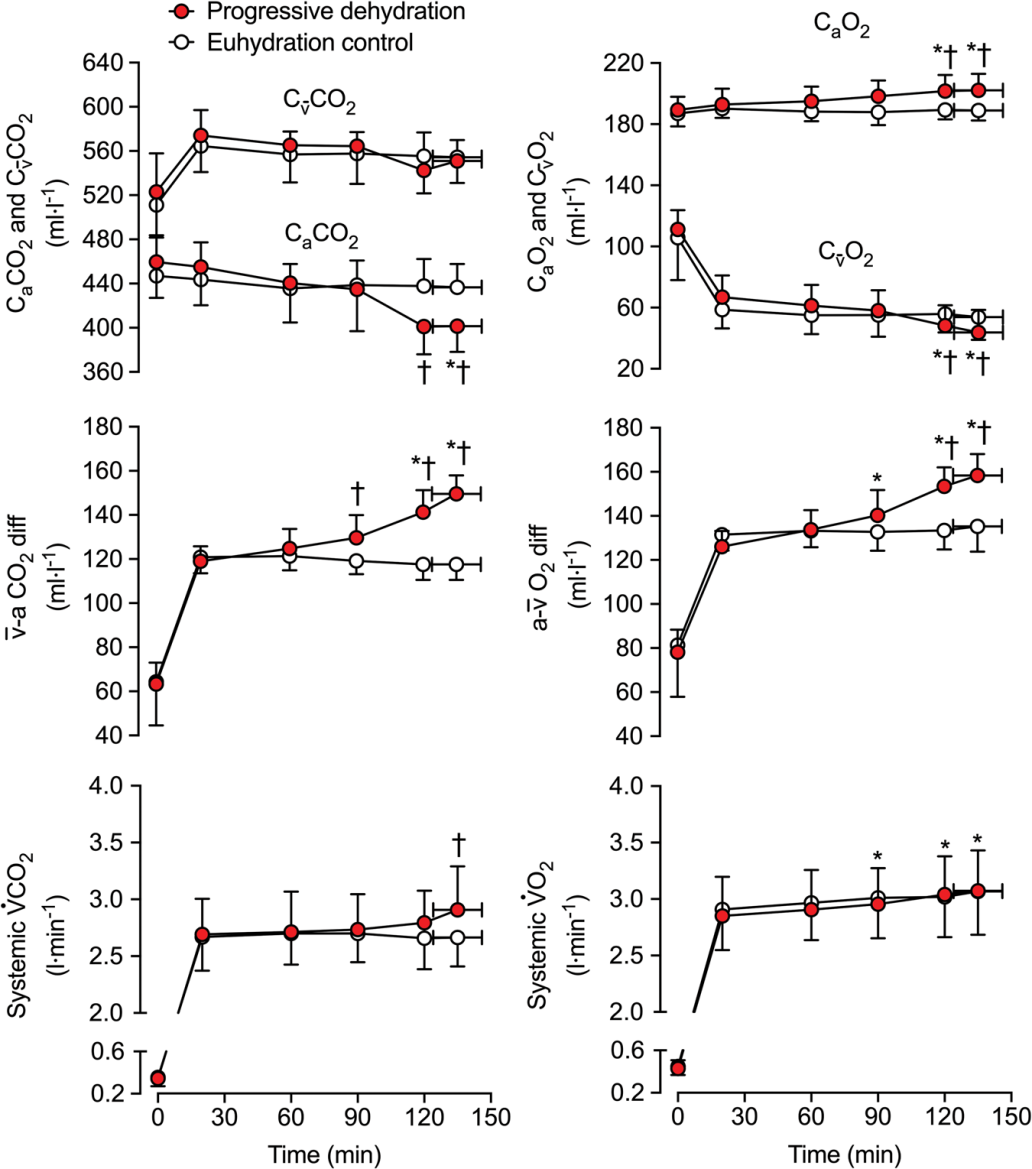
Pulmonary gas exchange during prolonged, intense exercise with varied hydration status. C_a_CO_2_, C_v_CO_2_,  $\bar{v}$–aCO_2_ diff, pulmonary ${\dot{V}}_{{\mathrm{CO}}_{2}}$, C_a_O_2_, C_v_O_2_, a–$\bar{v}$O_2_ diff and pulmonary ${\dot{V}}_{O_{2}}$ during the progressive dehydration and euhydration control trials. Data are shown as mean ± SD for 7 subjects. ^*^Significantly different from 20 min, *P* < 0.05. ^†^Significantly different from euhydration control, *P* < 0.05

During the 20–135 ± 11 min of exercise in the dehydration and hyperthermia trial, ${\dot{V}}_{E}$ and *f*_b_ increased by 19 ± 8 L min^−1^ (*P* = 0.006) and 7 ± 4 breaths min^−1^ (*P =* 0.003), respectively, whereas the increase was of 6 ± 2 L min^−1^ (*P =* 0.003) and 4 ± 2 breaths min^−1^ (*P =* 0.044) in control conditions. As a result, at end of exercise, ${\dot{V}}_{E}$ and *f*_b_ were 13 ± 8 L min^−1^ (*P* = 0.004) and 7 ± 4 breaths min^−1^ (*P =* 0.004) higher, respectively, in the dehydration and hyperthermia trial. In contrast, *V*_T_ remained unchanged over time (*P* = 0.822) and was not different between trials (*P* = 0.0599; Figure 1). The *V*_D_/*V*_T_ was maintained constant throughout the exercise bouts (*P =* 0.589) and did not differ between the trials (*P =* 0.235). The ${\dot{V}}_{A}$ increased more in the dehydration and hyperthermia trial compared with the control trial (*P* < 0.001), whereas cardiac output was reduced by 3.3 ± 1.5 L min^−1^ (*P =* 0.001); thus, the ventilation to perfusion ratio (${\dot{V}}_{A}$/$\dot{Q}$) was elevated in the dehydration and hyperthermia trial compared with the control trials after 120 min of exercise (*P <* 0.012; Figure 1). Upon completion of exercise in the dehydration and hyperthermia trial, $P_{\mathrm{ETC}O_{2}}$ and arterial [HCO_3_^−^] were reduced (*P* ≤ 0.031; Table 2; Figure 2), and $P_{\mathrm{aC}O_{2}}$ was 4 ± 3 mmHg lower than control (*P =* 0.006; Figure 2). Conversely, the arterial partial pressure of oxygen ($P_{aO_{2}}$) was 11 ± 6 mmHg higher at the end of exercise in the dehydration and hyperthermia trial (*P =* 0.002; Figure 2), and end‐tidal partial pressure of O_2_ ($P_{\mathrm{ET}O_{2}}$) and arterial pH (pH_a_) were elevated (*P* ≤ 0.035; Table 2; Figure 2). Correspondingly, mixed‐venous to arterial carbon dioxide difference ($\bar{v}$–aCO_2_ diff) and arterial to mixed‐venous oxygen difference (a–$\bar{v}$O_2_ diff) were 32 ± 7 ml L^−1^ (*P =* 0.001) and 23 ± 11 ml L^−1^ (*P <* 0.001) higher, respectively, in the dehydration and hyperthermia trial compared with the control trial (Figure 3).

**FIGURE 4:**
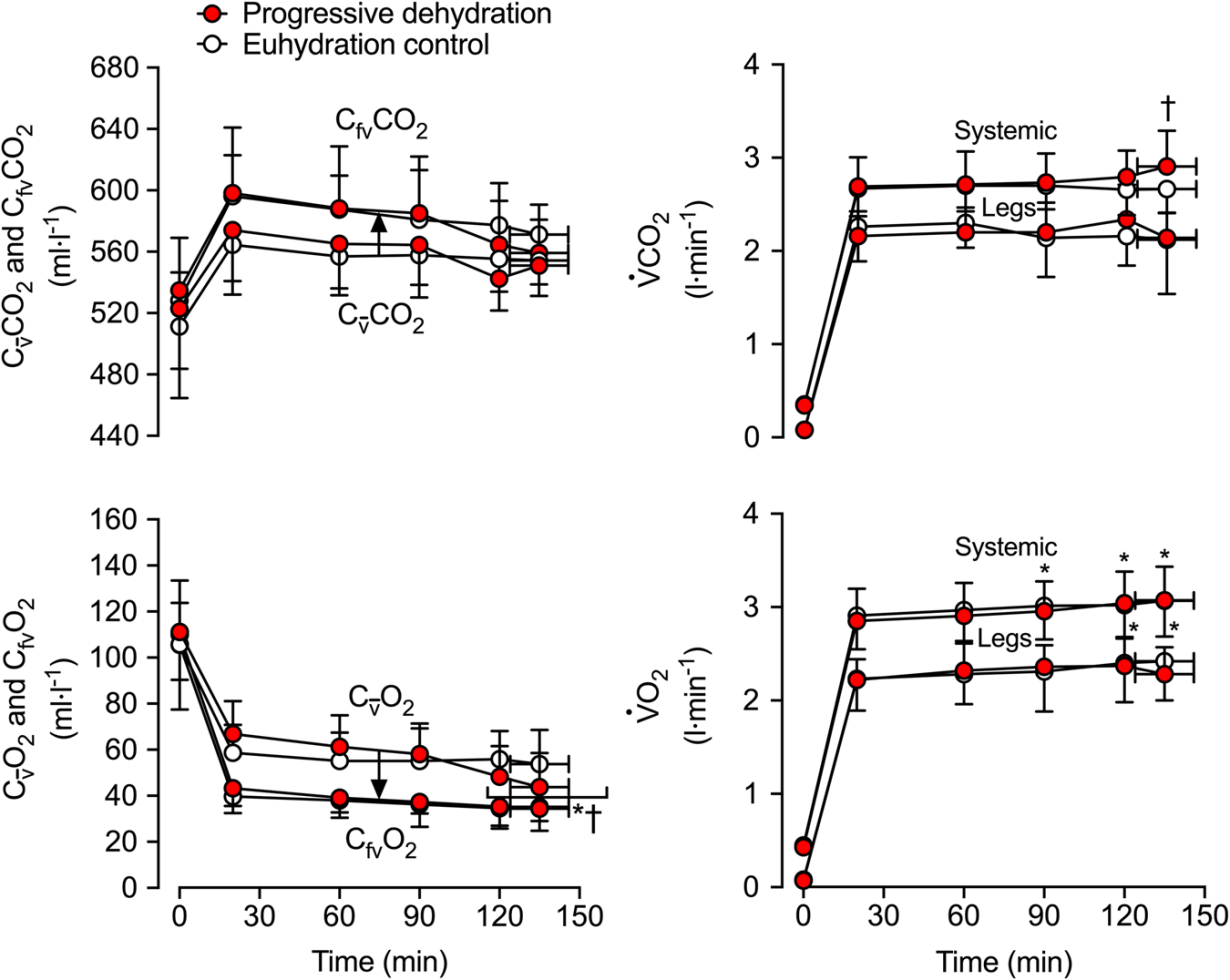
Venous blood gas content across the pulmonary circulation and the exercising leg and the corresponding pulmonary and two‐legged ${\dot{V}}_{CO_{2}}$ and ${\dot{V}}_{O_{2}}$ at rest and during prolonged, intense exercise with varied hydration status. C_fv_CO_2_, C$\bar{v}$CO_2_, C_fv_O_2_, C$\bar{v}$O_2_, pulmonary ${\dot{V}}_{CO_{2}}$, legs ${\dot{V}}_{CO_{2}}$, pulmonary ${\dot{V}}_{O_{2}}$ and legs ${\dot{V}}_{O_{2}}$ during the progressive dehydration and euhydration control trials. Data are shown as mean ± SD for 7 subjects. ^*^Significantly different from 20 min, *P* < 0.05. ^†^Significantly different from euhydration control, *P* < 0.05. C$\bar{v}$O_2_ declined at 120 and 134 min compared to 20 min of the progressive dehydration trial, with values being lower than in the euhydration control trial

The $S_{aO_{2}}$, ABE, $C_{\mathrm{fv}O_{2}}$, $C_{\mathrm{fvC}O_{2}}$ and $C_{\bar{v}{\mathrm{CO}}_{2}}$ did not change during exercise (*P ≥* 0.142) or between hydration conditions (all *P* ≥ 0.151; Table 2; Figures 2, 3, 4). In contrast, $C_{\mathrm{aC}O_{2}}$ declined after 2 h of exercise (*P <* 0.014) in the dehydration and hyperthermia trial, and arterial oxygen content ($C_{aO_{2}}$) was elevated after 2 h of exercise (*P =* 0.039). Both $C_{\mathrm{aC}O_{2}}$ and $C_{aO_{2}}$ remained unchanged throughout the control trial (*P >* 0.200; Figure 3). The increase in $\bar{v}$–aCO_2_ diff with dehydration and hyperthermia was associated solely with a decline in $C_{\mathrm{aC}O_{2}}$, whilst the rise in a–$\bar{v}$O_2_ diff was associated with both an increase in $C_{aO_{2}}$ and a reduction in $C_{\bar{v}O_{2}}$ (Figure 3). In the dehydration and hyperthermia trial, arterial noradrenaline concentration ([NA]_a_) and arterial adrenaline concentration ([A]_a_) were significantly higher than control values after 60 and 90 min of exercise, respectively (*P* ≤ 0.045; Table 2). Pulmonary ${\dot{V}}_{CO_{2}}$ was elevated above control values at the end of exercise in the dehydration and hyperthermia trial (*P =* 0.005), whereas ${\dot{V}}_{O_{2}}$ increased to the same extent in both trials from 90 min onwards (*P <* 0.016; Figure 3). Similar time‐dependent responses were observed in both trials for pulmonary mixed‐venous and femoral venous blood gases, and for ${\dot{V}}_{O_{2}}$ and ${\dot{V}}_{CO_{2}}$ across the legs and the lungs, except for $C_{\bar{v}O_{2}}$ and pulmonary ${\dot{V}}_{CO_{2}}$ values at exhaustion, which were reduced and elevated, respectively, with progressive dehydration compared with control values (Figure 4).

**FIGURE 5:**
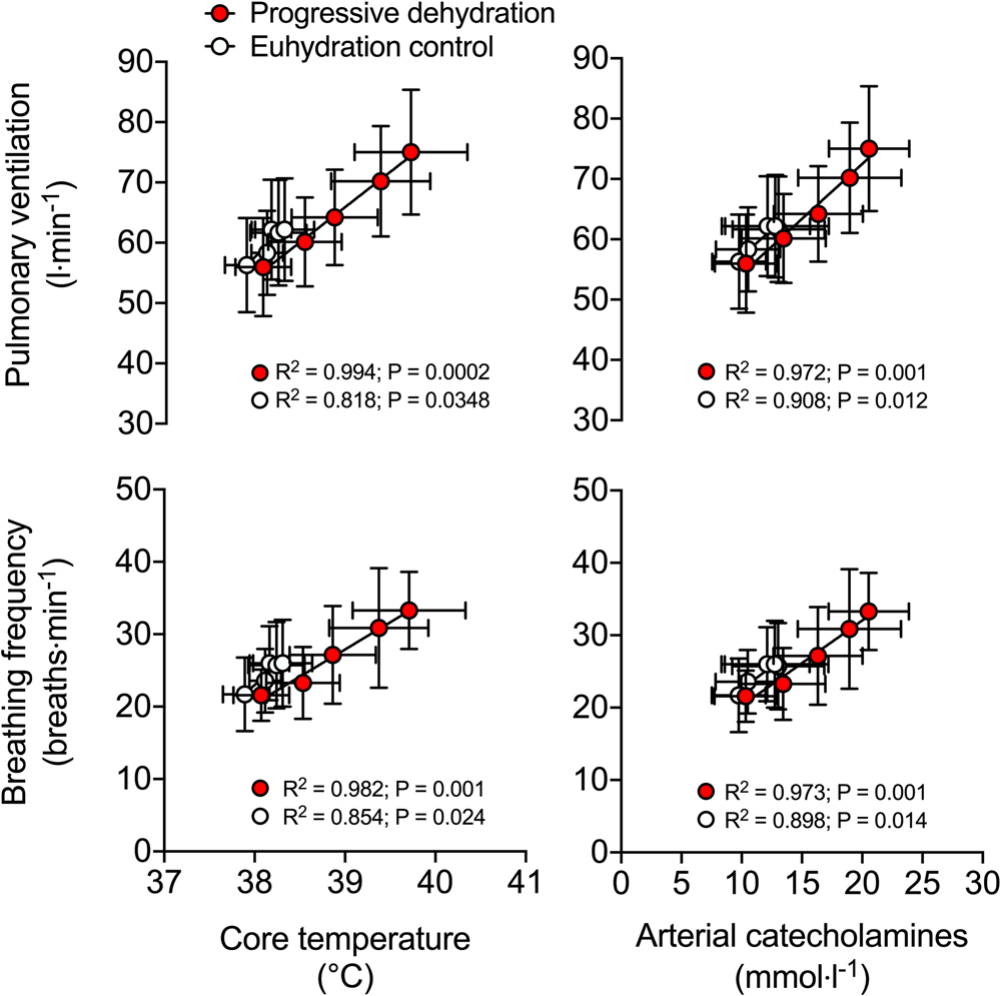
Relationships among ventilation, breathing frequency, core temperature and circulating catecholamines during prolonged, intense exercise with varied hydration status. Strong positive relationships were found between the elevations in ${\dot{V}}_{E}$ and *f*_b_ and the rise in T_c_ and [NA]_a_ in both trials. Exercise data are shown as mean ± SD for 7 subjects

The increase in ${\dot{V}}_{E}$ during exercise in the dehydration and control trials was closely related to the rise in: *T*_c_ (*R* ^2^ = 0.994, *P =* 0.0002 and *R* ^2^ = 0.818, *P =* 0.0348); [NA]_a_ (*R* ^2^ = 0.973, *P =* 0.001 and *R* ^2^ = 0.900, *P =* 0.013); [A]_a_ (*R* ^2^ = 0.933, *P =* 0.007 in the dehydration and hyperthermia trial only); and combined catecholamines (i.e., [NA] + [A]; *R* ^2^ = 0.972, *P =* 0.001 and *R* ^2^ = 0.908, *P =* 0.012; Figure 5). The slopes of the ${\dot{V}}_{E}$–*T*_c_ relationships were 12 ± 1 versus 15 ± 4 L min^−1^°C^−1^ in the dehydration and hyperthermia and control trials, respectively. The increase in *f*_b_ in the dehydration and control trials was also closely related to the elevations in *T*_c_ (*R* ^2^ = 0.982, *P =* 0.001 and *R* ^2^ = 0.854, *P =* 0.024); [NA]_a_ (*R* ^2^ = 0.972, *P =* 0.001 and *R* ^2^ = 0.890, *P =* 0.015); [A]_a_ (*R* ^2^ = 0.908, *P =* 0.012 in the dehydration and hyperthermia trial only); and combined catecholamines (*R* ^2^ = 0.973, *P =* 0.001 and *R* ^2^ = 0.898, *P =* 0.014; Figure 5). The slopes of the *f*_b_–*T*_c_ relationships were 8 ± 1 versus 11 ± 3 breaths min^−1^°C^−1^ in the dehydration and hyperthermia versus control trials.

### 3.2 Studies 2 and 3: Independent effects of hyperthermia and dehydration on the ventilatory response to prolonged exercise

**FIGURE 6:**
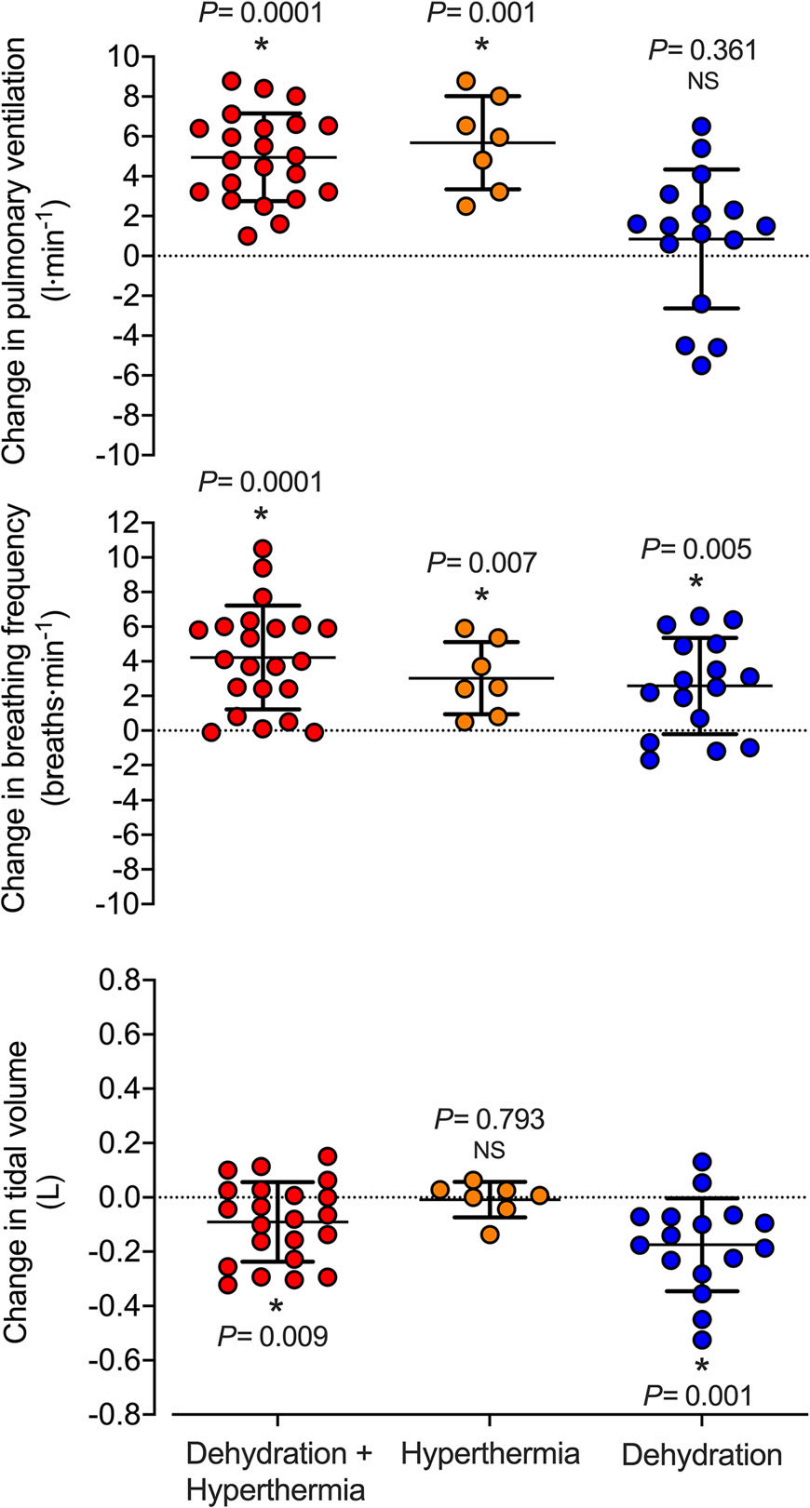
Changes in ventilatory responses to exercise with combined dehydration and hyperthermia, isolated hyperthermia and isolated dehydration compared to euhydration control. Changes in ventilation, respiratory frequency and tidal volume induced by hyperthermia and dehydration, hyperthermia alone and dehydration alone. Data are shown as mean ± SD for 22, 7 and 8 participants, respectively. The effects of combined dehydration and hyperthermia include additional data (*n* = 15) from two published studies with same experimental conditions (González‐Alonso, Mora‐Rodríguez *et al.* 1999 and 2000), whereas the effects of dehydration alone also illustrate data from the dehydration and blood volume restoration condition in the same individuals. ^*^Significant effect compared to the respective control condition, *P* < 0.05

During the 30 min exercise bouts, combined dehydration and hyperthermia (*T*_c_ +0.9 ± 0.2°C; 4.0 ± 0.5% body mass loss) increased ${\dot{V}}_{E}$ by 5.0 ± 2.2 L min^−1^ (*P =* 0.0001) and *f*_b_ by 4 ± 3 breaths min^−1^ (*P =* 0.0001), whereas *V*_T_ fell by 90 ± 150 ml (*P =* 0.009; Table 3; Figure 6). Hyperthermia alone (*T*_c_ +1.0 ± 03°C; 0.2 ± 0.3% body mass loss) increased ${\dot{V}}_{E}$ by 5.7 ± 2.3 L min^−1^ (*P =* 0.001) and *f*_b_ by 3 ± 2 breaths min^−1^ (*P =* 0.007) and reduced $P_{\mathrm{ETC}O_{2}}$ by 3 ± 2 mmHg (*P =* 0.006) without altering *V*_T_ (*P =* 0.793). In contrast, dehydration alone (*T*_c_ +0.1 ± 0.1°C; 4.1 ± 0.2% body mass loss) did not alter ${\dot{V}}_{E}$ (*P =* 0.361) or $P_{\mathrm{ETC}O_{2}}$ (*P =* 0.899), but reduced *V*_T_ (by 175 ± 200 ml; *P =* 0.001) and increased *f*_b_ (by 2.4 ± 3.0 breaths min^−1^; *P =* 0.005; Table 3).

**TABLE 3:** Thermal, body fluid and respiratory responses to 30 min of intense exercise with dehydration combined with hyperthermia, isolated hyperthermia, isolated dehydration, and dehydration with blood volume restoration, compared with corresponding responses in euhydration control trials

	**Combined effect of dehydration and hyperthermia**		**Isolated effect of hyperthermia**		**Isolated effect of dehydration**		**Effect of dehydration and BV restoration**	
**Variables**	**Control**	**Dehydration + Hyperthermia**	**Control**	**Hyperthermia**	**Control**	**Dehydration**	**Control**	**Dehydration + BVR**
*T*_c_, °C	38.2 ± 0.4	39.1 ± 0.4*	38.3 ± 0.2	39.2 ± 0.3*	38.1 ± 0.4	38.2 ± 0.3	38.0 ± 0.4	38.1 ± 0.3
*T*_sk_, °C	34.2 ± 0.6	34.7 ± 0.9*	34.0 ± 0.6	34.6 ± 0.8*	21.2 ± 1.3	20.4 ± 1.2	20.9 ± 1.4	20.9 ± 0.9
Body mass loss, %	−0.5 ± 0.3	−4.6 ± 0.5*	0.0 ± 0.1	−0.2 ± .03	−0.1 ± 0.2	−4.2 ± 0.3*	−0.3 ± 0.2	−4.2 ± 0.4*
[Haemoglobin], g L^−1^	159 ± 8	168 ± 10*	159 ± 6	160 ± 7	156 ± 8	164 ± 7*	156 ± 8	153 ± 9*
Osmolality, mosmol kg^−1^	278 ± 4	298 ± 4*	279 ± 3	281 ± 2	281 ± 3	296 ± 5*	281 ± 3	296 ± 5*
${\dot{V}}_{O_{2}}$, L min^−1^	3.09 ± 0.25	3.10 ± 0.26	3.15 ± 0.28	3.16 ± 0.27	3.22 ± 0.34	3.20 ± 0.34	3.21 ± 0.34	3.22 ± 0.33
${\dot{V}}_{CO_{2}}$, L min^−1^	2.94 ± 0.26	2.97 ± 0.26	3.05 ± 0.29	3.02 ± 0.21	3.04 ± 0.34	2.99 ± 0.31	3.00 ± 0.33	2.95 ± 0.31*
${\dot{V}}_{E}$, L min^−1^	68.6 ± 6.2	73.5 ± 6.9*	68.5 ± 4.9	74.2 ± 6.6*	67.5 ± 5.8	68.1 ± 5.5	65.5 ± 6.0	66.6 ± 4.9
*f*_b_, breaths min^−1^	34 ± 6	38 ± 7*	35 ± 6	38 ± 6*	30 ± 4	32 ± 4	30 ± 5	33 ± 3*
*V*_T_, L	2.05 ± 0.24	1.96 ± 0.26*	2.00 ± 0.25	1.99 ± 0.24	2.31 ± 0.47	2.14 ± 0.32*	2.25 ± 0.46	2.08 ± 0.37*
$P_{\mathrm{ETC}O_{2}}$, mmHg	37 ± 4	35 ± 4	38 ± 4	35 ± 3*	39 ± 6	39 ± 4	39 ± 4	39 ± 3

*Note*: Values are means ± SD for participants in the combined dehydration and hyperthermia (*n* = 22), the hyperthermia alone (*n* = 7) (study 2) and the dehydration alone and dehydration with blood volume restoration (*n* = 8) (study 3) studies, respectively. The effects of combined dehydration and hyperthermia include additional data (*n* = 15) from two published studies with the same experimental conditions and experimental protocol (González‐Alonso, Mora‐Rodríguez et al., 1999; González‐Alonso et al., 2000).

Abbreviations: BVR, blood volume restoration ; *f*_b_, breathing frequency; $P_{\mathrm{ETC}O_{2}}$, end‐tidal partial pressure of CO_2_; *T*_c_, core temperature; *T*_sk_, mean skin temperature; ${\dot{V}}_{CO_{2}}$, pulmonary CO_2_ production; ${\dot{V}}_{E}$, minute ventilation; ${\dot{V}}_{O_{2}}$, pulmonary O_2_ consumption; V_T_, tidal volume.

* Significantly different from the corresponding control conditions, *P <* 0.01.

Blood volume restoration in dehydrated and normothermic individuals did not alter the ventilatory responses to exercise. Indeed, as observed with isolated dehydration, ${\dot{V}}_{E}$ and $P_{\mathrm{ETC}O_{2}}$ remained unchanged compared with control conditions (i.e., euhydrated normothermic; *P =* 0.369 and *P =* 0.836, respectively), *f*_b_ increased (by 3 ± 3 breaths min^−1^, *P =* 0.039) and *V*_T_ declined (by 174 ± 171 ml, *P =* 0.013; Table 3). When both isolated dehydration and dehydration with blood volume restoration conditions were combined, ${\dot{V}}_{E}$ and $P_{\mathrm{ETC}O_{2}}$ were unchanged compared with control conditions (*P =* 0.361 and *P =* 1.000, respectively), but *f*_b_ increased (by 3 ± 3 breaths min^−1^, *P =* 0.005) and *V*_T_ declined (by 174 ± 171 ml, *P =* 0.001).

### 3.3 Study 4: Impact of adrenaline infusion on the ventilatory response to prolonged exercise in the heat

The plasma concentration of catecholamines increased progressively over time during the control exercise bout with saline infusion. Plasma adrenaline increased ∼2.5‐fold from 30 to 120 min of exercise (from 0.5 ± 0.2 to 1.2 ± 0.5 nmol L^−1^; *P =* 0.022), while plasma noradrenaline increased ∼2.1‐fold during the same period (from 6.3 ± 2.7 to 13.4 ± 6.6 nmol L^−1^; *P =* 0.042; Table 4). Adrenaline infusion significantly increased the plasma adrenaline concentration (*P* ≤ 0.013 vs. control), with average values of 7 and 8 nmol L^−1^ recorded at 60 and 120 min of exercise, respectively, while the plasma noradrenaline concentration was not different from control (*P =* 0.638; Table 4). The plasma concentration of combined catecholamines increased over time in both trials (*P =* 0.001) but was 6.5 ± 3.7 and 7.2 ± 4.3 nmol L^−1^ higher during adrenaline infusion at 60 and 90 min of exercise, respectively, when compared with control conditions (*P* ≤ 0.004).

**TABLE 4:** Thermal, metabolic and blood variables during prolonged intense exercise with adrenaline or saline infusion

		**Time (min)**			
**Variable**	**Trial**	**10–30**	**40–60**	**85–90**	**120**
*T*_c_, °C	Adrenaline infusion	37.8 ± 0.2	38.4 ± 0.2*^†^	38.6 ± 0.3*^†^	38.9 ± 0.2*^†^
	Saline infusion	37.8 ± 0.1	38.0 ± 0.2*	38.3 ± 0.2*	38.5 ± 0.1*
*T*_sk_, °C	Adrenaline infusion	33.5 ± 0.9	33.1 ± 0.8	32.9 ± 0.6	32.9 ± 0.8
	Saline infusion	33.6 ± 0.7	33.4 ± 0.8	33.2 ± 0.7	33.2 ± 0.6
${\dot{V}}_{O_{2}}$, L min^−1^	Adrenaline infusion	2.75 ± 0.14	2.84 ± 0.15*	2.95 ± 0.24*	3.03 ± 0.23*
	Saline infusion	2.77 ± 0.17	2.91 ± 0.19*	2.98 ± 0.24*	3.02 ± 0.24*
${\dot{V}}_{CO_{2}}$, L min^−1^	Adrenaline infusion	2.58 ± 0.14	2.76 ± 0.16*^†^	2.78 ± 0.20*^†^	2.84 ± 0.14*^†^
	Saline infusion	2.58 ± 0.14	2.67 ± 0.18	2.70 ± 0.21	2.74 ± 0.21
$P_{\mathrm{ETC}O_{2}}$, mmHg	Adrenaline infusion	43 ± 5	38 ± 5*^†^	37 ± 5*	36 ± 6*
	Saline infusion	41 ± 4	41 ± 4	38 ± 5	36 ± 7
[NA], nmol L^−1^	Adrenaline infusion	6.4 ± 2.8	7.5 ± 3.6	10.2 ± 5.2*	11.6 ± 5.6*
	Saline infusion	6.3 ± 2.7	7.6 ± 3.2	10.2 ± 4.7	13.4 ± 6.6
[A], nmol L^−1^	Adrenaline infusion	0.5 ± 0.2	7.1 ± 3.2*^†^	8.0 ± 3.9*^†^	7.2 ± 4.5*^†^
	Saline infusion	0.5 ± 0.2	0.5 ± 0.2	0.8 ± 0.3	1.2 ± 0.5*

*Note*: Values are means ± SD for seven participants in study 4.

Abbreviations: [A], adrenaline concentration; [NA], noradrenaline concentration; $P_{\mathrm{ETC}O_{2}}$, end‐tidal partial pressure of CO_2_; *T*_c_, core temperature; *T*_sk_, mean skin temperature.

* Significantly different from 10–30 min, *P <* 0.05.

^†^Significantly different from saline infusion, *P <* 0.05.

After 30 min of exercise, *T*_c_ was the same (i.e., 37.8 ± 0.1°C) in both trials and increased progressively throughout the remainder of exercise during each trial. During adrenaline infusion, *T*_c_ began to increase shortly after the start of the infusion, and by 40 min of exercise (i.e., 10 min postinfusion), *T*_c_ was 0.3 ± 0.2°C higher than during saline infusion (*P <* 0.001). Thereafter, *T*_c_ remained 0.3–0.4 ± 0.2°C higher during the adrenaline versus saline infusion trial (*P* ≤ 0.013), with values at 120 min of exercise of 38.9 ± 0.2 versus 38.5 ± 0.2°C, respectively (*P =* 0.007). However, *T*_sk_ and the dehydration level were similar in both trials (i.e., 2.9 ± 0.6 and 2.9 ± 0.4% body mass loss at the end of the 2 h of exercise in the saline and adrenaline infusion trials, respectively; *P >* 0.874; Table 4).

**FIGURE 7:**
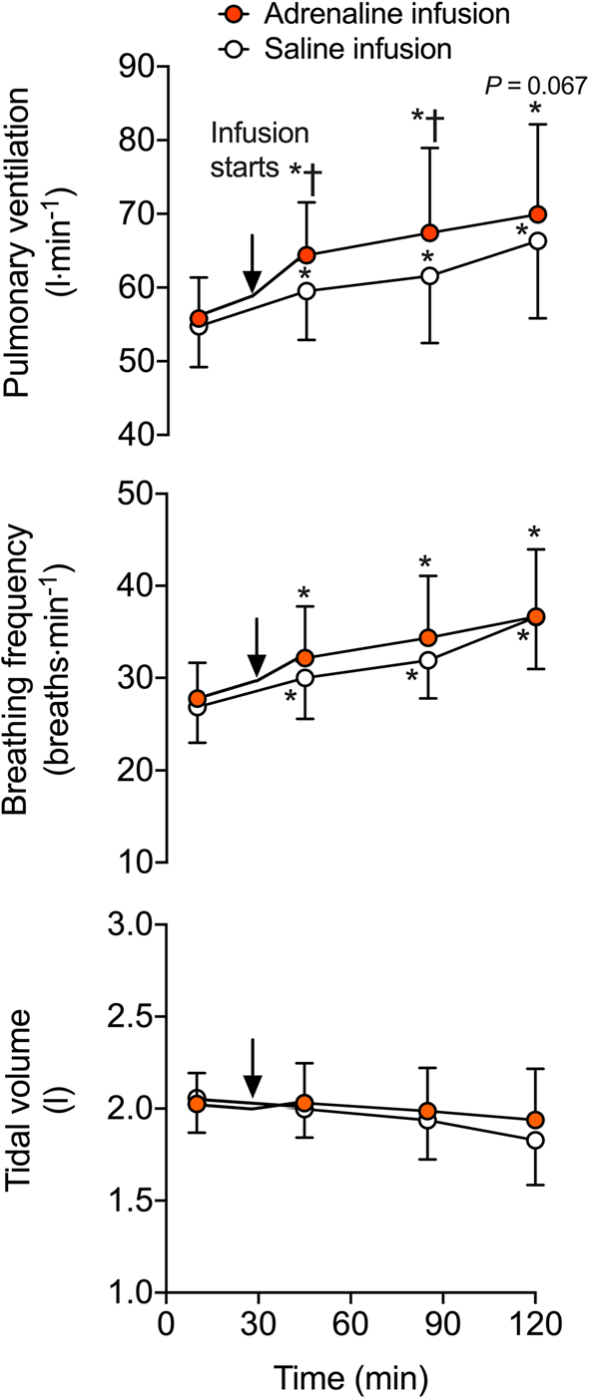
Ventilatory responses to prolonged exercise‐induced dehydration with adrenaline or saline infusion. Pulmonary ventilation, breathing frequency and tidal volume during the progressive dehydration with adrenaline or saline infusion. Data are shown as mean ± SD for 7 subjects. ^*^Significantly different from 10 min, *P* < 0.05. ^†^Significantly different from saline infusion, *P* < 0.05

The ${\dot{V}}_{E}$ was similar in both trials before infusion (i.e., 55.8 ± 2.1 L min^−1^ at 10 min of exercise; *P =* 0.170) and increased progressively throughout exercise in both trials (*P <* 0.001; Figure 7). However, soon after adrenaline infusion, ${\dot{V}}_{E}$ increased more rapidly, with ${\dot{V}}_{E}$ of 59.5 ± 6.6 versus 64.4 ± 7.2 L min^−1^ at 40 min in the saline and adrenaline infusion trials, respectively (*P =* 0.006). This ∼8% difference in ${\dot{V}}_{E}$ was maintained for the remainder of exercise, with final values of 66.3 ± 4.0 L min^−1^ in the saline trial versus 70.0 ± 4.6 L min^−1^ in the adrenaline trial (*P =* 0.067). The *f*_b_ progressively increased during exercise (*P <* 0.001), but no statistical differences were observed between trials (*P* = 0.121; Figure 7). The *V*_T_ did not change over time (*P =* 0.147) or between trials (*P =* 0.289; Figure 7). From a similar value of 2.76 ± 0.06 L min^−1^ at 10 min of exercise (i.e., pre‐infusion), ${\dot{V}}_{O_{2}}$ increased by 270 ml min^−1^ (8.6%) at the end of exercise in both trials (*P* ≤ 0.049; Table 4). Pre‐infusion, ${\dot{V}}_{CO_{2}}$ values were also similar between trials (average 2.58 ± 0.05 L min^−1^). After 90 min of exercise, ${\dot{V}}_{CO_{2}}$ was ∼4% higher in the adrenaline compared with the saline infusion trial (*P* ≤ 0.040), with the difference remaining unchanged until completion of the exercise test (Table 4). From 60 min onwards, $P_{\mathrm{ETC}O_{2}}$ was reduced and maintained below the 30 min value during the adrenaline infusion trial only (*P* ≤ 0.010; Table 4).

**FIGURE 8:**
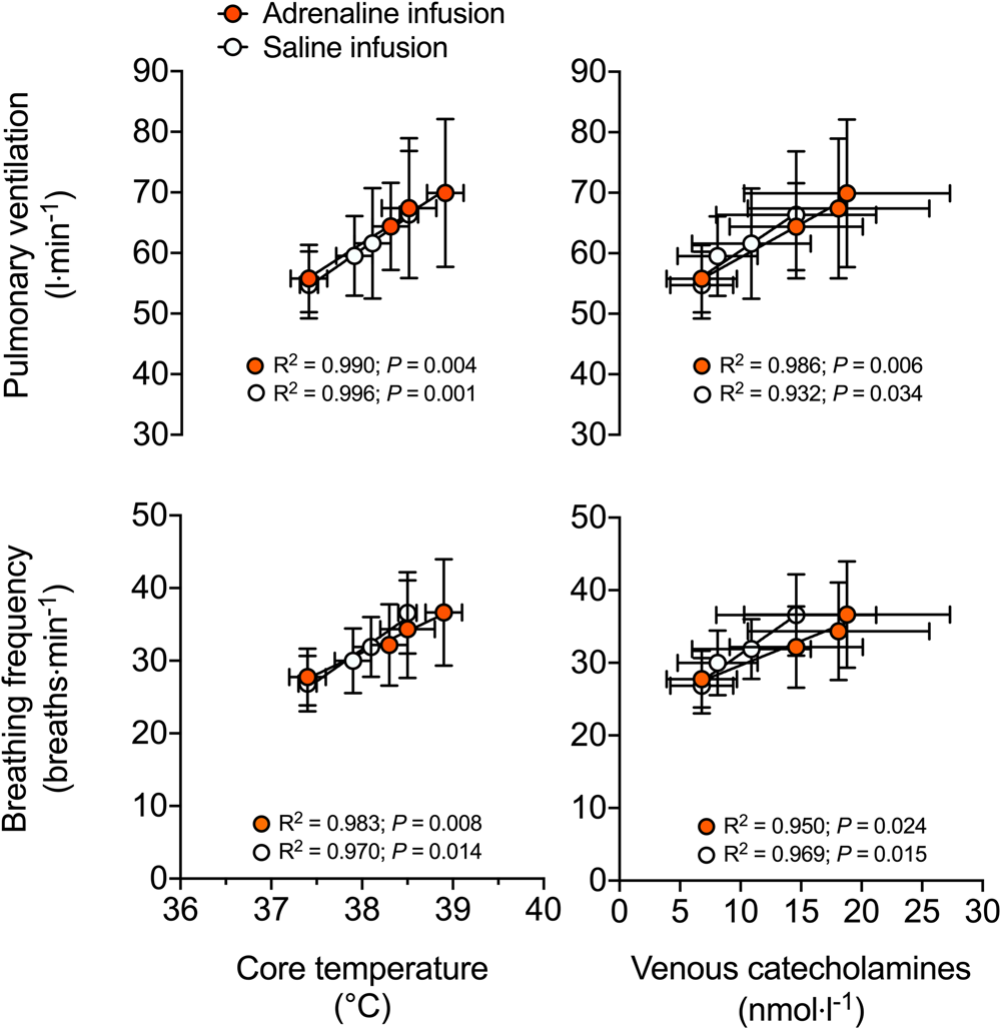
Relationships among ventilation (${\dot{V}}_{E}$), breathing frequency (*f*_b_), core temperature (*T*_c_) and circulating catecholamines during prolonged intense exercise with adrenaline or saline infusion. Strong positive relationships were found between the elevations in ${\dot{V}}_{E}$ and *f*_b_ and the rise in *T*_c_ and [catecholamines] in both trials. Exercise data are shown as the mean ± SD for seven subjects

The increase in ${\dot{V}}_{E}$ during exercise in the adrenaline and saline infusion trials was closely related to the rise in: *T*_c_ (*R* ^2^ = 0.990, *P =* 0.004 and *R* ^2^ = 0.996, *P =* 0.001); [NA]_a_ (*R* ^2^ = 0.973, *P =* 0.001 and *R* ^2^ = 0.900, *P =* 0.013); and combined catecholamines (*R* ^2^ = 0.986, *P =* 0.006 and *R* ^2^ = 0.932, *P =* 0.034; Figure 8). The slopes of the ${\dot{V}}_{E}$–*T*_c_ relationships were 10 ± 1 and 11 ± 1 L min^−1^°C^−1^ in the adrenaline and saline infusion trials, respectively. The increase in *f*_b_ in the adrenaline and saline infusion trials was also closely related to the increases in: *T*_c_ (*R* ^2^ = 0.983, *P =* 0.008 and *R* ^2^ = 0.970, *P =* 0.014); [NA]_a_ (*R* ^2^ = 0.972, *P =* 0.001 and *R* ^2^ = 0.890, *P =* 0.015); and combined catecholamines (*R* ^2^ = 0.950, *P =* 0.024 and *R* ^2^ = 0.969, *P =* 0.015; Figure 8). The slopes of the *f*_b_–*T*_c_ relationships were 6 ± 1 and 9 ± 1 breaths min^−1^°C^−1^ in the adrenaline and saline infusion trials, respectively.

## 4 DISCUSSION

When people become dehydrated and hyperthermic through prolonged exercise, they hyperventilate. This investigation established that hyperventilation during prolonged intense exercise is intimately related to elevations in core temperature and circulating catecholamines and that compensatory adjustments in pulmonary CO_2_ and O_2_ exchange accompanied the blunting of pulmonary and systemic blood flow associated with dehydration and hyperthermia, such that ${\dot{V}}_{CO_{2}}$ increased and ${\dot{V}}_{O_{2}}$ remained unchanged. Moreover, we found that isolated hyperthermia increased ${\dot{V}}_{E}$ to similar levels as combined dehydration and hyperthermia and that preventing hyperthermia in dehydrated individuals restored ${\dot{V}}_{E}$ to euhydration control levels. Isolated dehydration therefore did not lead to hyperventilation. Finally, adrenaline infusion during exercise elevated core temperature and ${\dot{V}}_{E}$, suggesting adrenergic modulation of breathing during prolonged intense exercise. Overall, these findings support the idea that hyperthermia is responsible for the excess rise in ventilation during endurance exercise and that adjustments in pulmonary gas exchange occur when prolonged exercise is associated with progressive dehydration and hyperthermia.

### 4.1 Pulmonary ventilation and gas exchange during endurance exercise

A new and important finding of this investigation is that, when humans perform endurance exercise and become progressively dehydrated and hyperthermic, gas exchange adjustments occur at both pulmonary and tissue levels, meaning that peripheral tissue and organ oxygenation and blood acid–base homeostasis are largely preserved, despite the blunting of pulmonary and systemic circulations. On the one hand, the increase in blood oxygenation (∼11 mmHg in $P_{aO_{2}}$ and ∼12 ml L^−1^ in $C_{aO_{2}}$) in combination with the increase in O_2_ extraction from the circulation by active muscles and other bodily tissues (∼23 ml L^−1^ in a–$\bar{v}$O_2_ diff and ∼7% elevation in systemic O_2_ extraction) fully compensated for the reduced cardiac output (of ∼3.3 L min^−1^), such that ${\dot{V}}_{O_{2}}$ was maintained in our dehydrated and hyperthermic participants. On the other hand, the enhanced pulmonary ${\dot{V}}_{CO_{2}}$, which happened despite a reduced pulmonary circulation, was solely associated with a decline in arterial CO_2_ content (∼4 mmHg in $P_{\mathrm{aC}O_{2}}$ and ∼35 ml L^−1^ in $C_{\mathrm{aC}O_{2}}$) that accounted entirely for the increase in $\bar{v}$–aCO_2_ diff (∼32 ml L^−1^; Figures 2 and 3).

Theoretically, there are at least three possibilities to explain the present observations as follows: (1) a diminished pulmonary blood flow could have led to a prolonged pulmonary transfer time, thereby facilitating gas exchange between the alveoli and the pulmonary capillaries and vice versa; (2) an increased blood temperature could have facilitated the unloading of O_2_ to the active tissues; or (3) a combination of both. Although pulmonary transit time can reduce towards the minimum time required for alveolar–arterial $P_{O_{2}}$ equilibration in the high‐intensity exercise domain (80–100% of ${\dot{V}}_{O_{2}\max}$) and might therefore be implicated in some of the gas exchange abnormalities reported in population groups similar to the one studied here [i.e., well‐trained, young male athletes (Dempsey et al., 1984; Nielsen et al., 1998)], the time for alveolar–arterial $P_{O_{2}}$ equilibration is usually deemed sufficient at lower exercise intensities (such as used in this study). To support this assertion, none of the participants developed arterial oxygen desaturation in either the dehydration or the control conditions. To understand the effect of reduced cardiac output on pulmonary gas exchange, we applied the Piiper and Scheid (1981) model for capillary–alveolar equilibration to quantify the level of diffusion/perfusion limitation via the ‘*Y*’ parameter. A ‘*Y*’ value > 3 indicates pure perfusion limitation, whereas ‘*Y*’ < 0.1 indicates pure diffusion limitation. Values of ‘*Y*’ between 0.1 and 3 indicate mixed perfusion–diffusion limitation. The estimated mean ‘*Y*’ values for the dehydrated and euhydrated conditions were 1.75 and 1.54, respectively. This suggests a slightly higher level of O_2_ diffusion limitation in the euhydrated conditions, implying that the reduction of cardiac output with dehydration offset the negative impact of hyperthermia on lung O_2_ diffusion.

As expected, alveolar hyperventilation resulted in increased pH_a_ and decreased [HCO_3_^−^]_a_, but the ABE_a_ remained unchanged. This is suggestive of minimal blood acid–base disturbances despite the exhaustive nature of the exercise and the alterations in locomotor muscle metabolism (González‐Alonso, Calbet et al., 1999). Furthermore, given that femoral venous $P_{O_{2}}$, oxyhaemoglobin and total O_2_ content were similar in the dehydration and hyperthermia trial compared with the euhydration control trial (Figure 4) and that jugular venous $P_{O_{2}}$, oxyhaemoglobin and total O_2_ content were found to be unaltered by dehydration and hyperthermia in another study (Trangmar et al., 2014), the effect of blood temperature on oxygen binding to haemoglobin is likely to have been negligible in these in vivo conditions. Collectively, the present arterial blood gas data therefore dispel the idea of a limiting role of pulmonary blood flow on gas exchange, blood oxygenation and blood CO_2_ content when fit young males perform prolonged intense exercise and become progressively dehydrated and hyperthermic.

The compensatory adjustments in pulmonary gas exchange and blood gas homeostasis were associated with increases in ventilation. Endurance exercise with combined dehydration and hyperthermia led to a higher ${\dot{V}}_{E}$ (of ∼13 L min^−1^ compared with control conditions), which was driven exclusively by an accelerated rate of breathing (∼7 breaths min^−1^; Figure 1). In a previous study conducted in healthy young males (Fujii et al., 2008b), two bouts of cycling [i.e., 30−60 min at 50% of peak oxygen uptake (${\dot{V}}_{O_{2}\mathrm{peak}}$) in a 35°C environment, with 70–80 min rest in between], which led to moderate dehydration (∼3.9% body mass loss), did not affect ventilation in healthy young males. The moderate exercise intensity was, however, associated with a similar final core temperature (∼38.2°C). Conversely, in the present study, the continuous, more intense and prolonged exercise led to a larger rise in *T*_c_ in the dehydration conditions (∼39.7°C), resulting in a ∼1.5°C difference in *T*_c_ at the end of the trials. Hence, the increase in ${\dot{V}}_{E}$ in the present study closely followed the increase in *T*_c_ at a rate of 12–15 L min^−1^°C^−1^ in both sets of conditions (Figure 5). This ventilatory sensitivity to hyperthermia agrees fairly well with previous findings in young male adults (i.e., ${\dot{V}}_{E}$ increase of ∼5–12 L min^−1^°C^−1^; Fujii et al., 2008a, 2008b; Hayashi et al., 2006, 2009; Nybo & Nielsen, 2001; Tsuji et al., 2012), as does the concomitant increase in breathing frequency with body core temperature (8–11 breaths min^−1^°C^−1^; Chu et al., 2007; Fujii et al., 2008a, 2011; Hayashi et al., 2011, 2006; Martin et al., 1979; Tsuji et al., 2012). Although these strong relationships suggest that hyperthermia might explain at least part of the ventilatory response in dehydrated humans, other factors known to influence ventilation, such as fatigue and increased plasma osmolality (up to ∼21 mosmol kg^−1^ or 7%), K^+^ (up to ∼0.4 mmol L^−1^ or 8%) and catecholamines (up to ∼8 mmol L^−1^ or 61%), were also present towards the end of prolonged exercise in the dehydration and hyperthermia trial, but absent in the control non‐exhaustive, iso‐time trial. Hence, to gain insight into the contributions of hydration and heat‐sensitive factors in increased ventilation during endurance exercise, we next evaluated the independent effects of dehydration and hyperthermia, without the confounding influences of fatigue, when blood osmolality and K^+^ were elevated but participants remained normothermic and when blood variables remained at control levels but hyperthermia was present.

### 4.2 Mechanisms of respiratory control during endurance exercise: Impact of dehydration, hyperthermia and adrenaline infusion

Two additional studies conducted during intense non‐exhaustive exercise afforded the identification of the isolated effects of dehydration and hyperthermia on ventilation. These studies demonstrated that: (1) the ventilatory response to endurance exercise does not differ between conditions of isolated hyperthermia (+1°C increase in *T*_c_ in euhydrated individuals) compared with combined hyperthemia and dehydration (+1°C increase in *T*_c_ and 4% body mass loss); that is, both conditions led to a rise in ${\dot{V}}_{E}$ of ∼5–6 L min^−1^, which was driven by an accelerated rate of breathing (∼3–4 breaths min^−1^), as in the first study; (2) isolated whole‐body dehydration did not increase ventilation above control levels (Figure 6); and (3) restoring blood volume with infusion of a plasma volume expander in dehydrated and normothermic individuals did not alter ventilation either. These observations point strongly to hyperthermia rather than dehydration‐related signals, such as hypovolaemia, hyperosmolality and hyperkalaemia (Paterson, 1997; Senay, 1969), as the principal stimulus increasing ventilation during endurance exercise in dehydrated and hyperthermic individuals. This raises the questions of which body temperature serves as the dominant thermal stimulus for the elevated ventilatory response (i.e., brain/spinal cord, core, skin and/or muscle temperature) and, by extension, whether hyperthermia increases *f*_b_ and ${\dot{V}}_{E}$ by acting directly on the central respiratory control system and/or indirectly via peripheral thermosensitive neurohumoral mechanisms.

In our experimental conditions, *T*_c_ was elevated by ∼1 or ∼1.5°C, while *T*_sk_ remained unchanged. Together with the intimate positive relationships between ${\dot{V}}_{E}$ and *T*_c_ and between *f*_b_ and *T*_c_ (Figure 5), these findings support the idea that internal body (not skin) temperature primarily drives hyperventilation during prolonged exercise. This agrees with previous findings displaying a linear relationship between ${\dot{V}}_{E}$ and *T*_c_ during mild and moderate‐intensity exercise, when variations in skin temperature (from 33 to 39°C) had little effect (Hayashi et al., 2006; Tsuji et al., 2012). Moreover, a recent study from our laboratory revealed that passive heating of human lower limbs, which induces a large rise in muscle temperature (from 32–34 to 38°C) but a small elevation in *T*_c_ (from 36.9 to 37.5°C), does not modify ${\dot{V}}_{E}$, *f*_b_ or *V*_T_ compared with control conditions (K. Watanabe, N. Koch Esteves, E. J. Stöhr, O. Gibson, K. Akiyama, S. Watanabe & J. González‐Alonso, unpublished observations). Activation of the muscle thermosensitive group III and IV afferents (Hertel et al., 1976; Kumazawa & Mizumura, 1977) might therefore have little involvement in hyperthermia‐induced hyperventilation during exercise (at least at muscle temperatures ≤38°C). Evidence in the isolated blood‐perfused lung also indicates that the normal lung is able to tolerate fairly severe blood temperature, ≤44°C (Cowen et al., 1992; Rickaby et al., 1991). This aligns with the classical observation that similar hyperthermia has no effect on cardiac output in the isolated mammalian heart (Knowlton & Starling, 1912). It therefore appears that, unlike the profound effects of local hyperthermia on the peripheral circulation (Koch Esteves et al., 2021), high lung, skin and muscle temperature per se might not play an obligatory role in the hyperventilation during endurance exercise.

An alternative possibility is that central temperature is the dominant thermal stimulus for hyperventilation during endurance exercise. In this context, increases in *T*_c_ could impact respiratory control mechanisms through changes in brain and spinal cord temperature. Animal studies have consistently shown that the spinal cord, hypothalamus and medulla oblongata are responsive to heat (Chai & Lin, 1972; Holmes et al., 1960; Tryba et al., 2003). In particular, increases in temperature within the physiological range (i.e., from ∼37 to 41°C) have been shown to increase activity of the respiratory pacemaker neurons located within the ventral respiratory group in the medulla oblongata (Tryba et al., 2003), hence to accelerate breathing frequency. Unlike animals that use panting as an evaporative heat‐loss mechanism (Robertshaw, 2006), the modification of breathing pattern in our study participants (i.e., accelerated rate of breathing) did not lead to a simultaneous increase in dead space (*V*_D_/*V*_T_ ratios remained constant at ∼0.13 from 20 min onwards in both experimental trials). Therefore, rather than a preferential ventilation of the upper airways and nasal passages that helps to dissipate heat, the tachypnoeic shift adopted by our hyperthermic participants led to alveolar hyperventilation and subsequent hypocapnia (with simultaneous declines in $P_{\mathrm{ETC}O_{2}}$, $P_{\mathrm{aC}O_{2}}$ and $C_{\mathrm{aC}O_{2}}$), increase in pH_a_ and reduction in [HCO_3_^−^]_a_. The progressive development of hypocapnia suggests that inhibitory feedback on ventilation owing to respiratory alkalosis (and central respiratory drive) was overridden by other factors and that the level of arterial H^+^ was not a primary determinant of the ventilatory response to exercise (as can happen in the higher‐intensity domain). Furthermore, given that carotid chemoreceptors are sensitive to decreases in $P_{aO_{2}}$ and pH and an increase in $P_{\mathrm{aC}O_{2}}$ (Kumar & Prabhakar, 2012), our blood gas data argue against carotid body stimulation as a key contributor to hyperthermic hyperventilation during exercise. Prior work that has used hyperoxic breathing during exercise to silence carotid chemoreceptors, and thereby attempt to evaluate the contribution of the carotid body chemoreflex to hyperthermic hyperventilation, has led to divergent findings (Fujii et al., 2019; Gibbons et al., 2022). It is, however, worth considering that, in addition to blood gas and pH stimuli, other blood‐borne physiochemical stimuli (including circulating catecholamines) can excite chemo‐afferent discharge of the carotid bodies (Kumar & Prabhakar, 2012) and that temperature itself can induce a heightened discharge of the chemoreceptor afferents (Gallego et al., 1979; McQueen & Eyzaguirre, 1974). Although the lack of effect of isolated dehydration rules out an obligatory role of hyperosmolality and hyperkalaemia, the intimate relationships between ${\dot{V}}_{E}$ (and *f*_b_) and circulating adrenaline, noradrenaline and combined catecholamines (Figure 5) support the idea that increases in sympathoadrenal activity (González‐Alonso et al., 1995; Ray, 1993; Saito et al., 1997) and/or efferent neural discharge to the respiratory muscles (Tryba et al., 2003) might be the mechanisms largely responsible for augmenting *f*_b_ and ${\dot{V}}_{E}$ when exercising humans become hyperthermic, regardless of the hydration status.

Adrenaline is often suggested as one of the possible feedforward factors involved in the control of breathing during exercise (Linton et al., 1992). In the last study, adrenaline infusion during exercise, which evoked a similar increase in catecholamines as in the first study (i.e., ∼7 mmol L^−1^), caused both rapid hyperthermia (*T*_c_ +0.4°C) and hyperventilation (${\dot{V}}_{E}$ +4 L min^−1^). Although these effects were sustained during the remaining 90 min of exercise, ${\dot{V}}_{E}$ and *f*_b_ kept increasing in association with the ensuing dehydration, hyperthermia and upward drift in catecholamines (Figure 7). To the best of our knowledge, limited research has investigated the effects of adrenaline infusion on ventilation (and its components, *f*_b_ or *V*_T_) during exercise in humans. In the two studies that reported ${\dot{V}}_{E}$ as supplementary data (Gaesser et al., 1994; Watt & Hargreaves, 2002), a ∼4 L min^−1^ additional rise in ${\dot{V}}_{E}$ was noted after adrenaline infusion in young active males performing submaximal cycling (i.e., 20 min at ∼69% of ${\dot{V}}_{O_{2}\mathrm{peak}}$ and 40 min at ∼59% of ${\dot{V}}_{O_{2}\mathrm{peak}}$, respectively), but failed to reach significance. In contrast, a significant difference in ${\dot{V}}_{E}$ (of ∼7 L min^−1^) was reported after adrenaline infusion when seven untrained males performed a 20 min exercise bout at a higher exercise intensity (i.e., power output initially corresponding to ∼77% of ${\dot{V}}_{O_{2}\mathrm{peak}}$; Womack et al., 1995). Although no simultaneous recordings of *T*_c_ were reported in those studies, the thermogenic effect of adrenaline in humans is well established, with vasoconstriction of the skin most likely to mediate this effect (Mora‐Rodríguez et al., 1996). Based on the strong associations between ${\dot{V}}_{E}$ (and *f*_b_) and circulating catecholamines (Figures 5 and 8), in addition to the comparable increases in ${\dot{V}}_{E}$ with isolated hyperthermia, combined dehydration and hyperthermia, and adrenaline infusion (i.e., ∼4–6 L min^−1^), we surmise that the effect of core body hyperthermia on ventilation can be mediated, at least in part, by elevated circulating catecholamines. From our understanding of the effects of catecholamines on carotid chemoreceptors, however, we do not exclude the possibility that a reflex mechanism triggered by adrenaline itself and/or adrenaline‐induced changes in plasma K^+^ might have contributed to an augmented ventilatory response (Joels & White, 1968; Linton et al., 1992). An implication of our findings is the possibility that, when humans sustain moderate‐ to heavy‐intensity activities over prolonged periods of time and become hyperthermic, increased levels of catecholamines modulate breathing.

### 4.3 Methodological considerations

Although minimizing biological variability by testing homogeneous samples of young, endurance‐trained participants has key advantages (Jager et al., 2017), we acknowledge that the inclusion of males only is a limitation of the present study. In this light, our findings await confirmation in females, because there is accumulating evidence of sex‐based differences in physiological responses to exercise [including ventilatory (Sheel, 2016) and sympathetic responses (Wheatley et al., 2014)]. A recent study comparing the responses of men and women during moderate exercise‐induced hyperthermia, however, found similar slopes of the ${\dot{V}}_{E}$ and core temperature relationship in men and women (Hayashi et al., 2012). We addressed the effects of sympathoadrenal activation on the ventilatory response to endurance exercise using i.v. infusion of adrenaline (study 4). This intervention emulated the sympathoadrenal hormonal milieu found during prolonged intense exercise inducing marked hyperthermia and dehydration (+7–8 nmol L^−1^ elevation in catecholamines; study 1; Table 4). To corroborate and extend our findings, future investigations should combine adrenaline and noradrenaline infusion (rather than adrenaline infusion alone), in addition to adrenergic receptor blockade (Heistad et al., 1972; Petersen et al., 1983; Wasserman et al., 1974). Finally, the ‘normal’ process of ageing leads to a wide range of physiological changes in the respiratory system that affect both the ventilatory response to exercise and the pulmonary circulation (Janssens, 2005; Taylor & Johnson, 2010). Although, in normal exercising conditions, arterial blood gases are generally well preserved in healthy older adults (Taylor & Johnson, 2010), more research is warranted to establish whether the homeostatic adjustments in blood oxygenation and acid–base balance observed in hyperthermic and dehydrated young adults occur in a similar manner in older populations subjected to heat stress.

### 4.4 Conclusion

**FIGURE 9:**
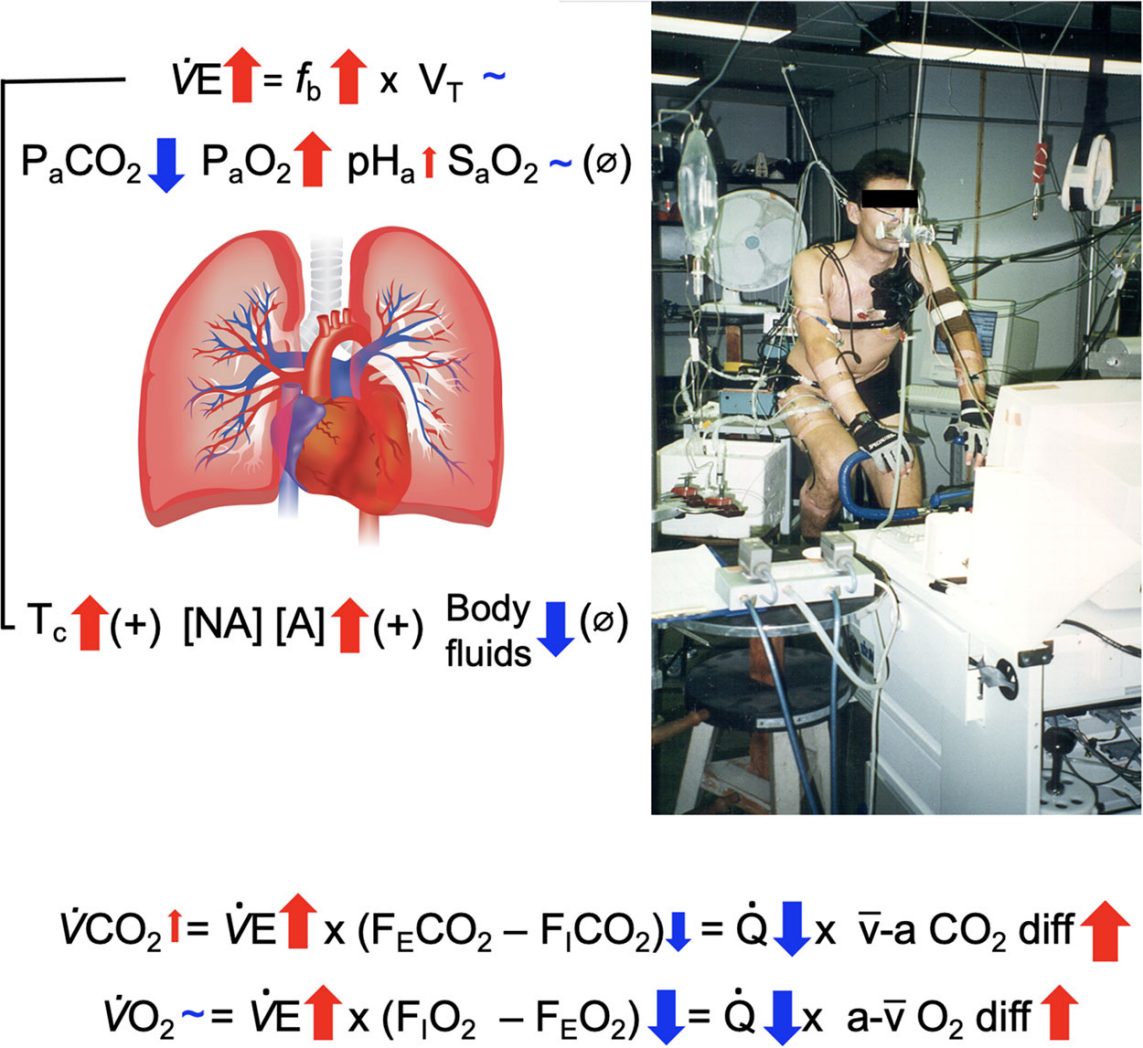
Schematic summary of the ventilatory and pulmonary gas exchange adjustments to prolonged exercise‐induced dehydration and hyperthermia and the potential contribution of increases in core temperature (*T*_c_) and sympathoadrenal activation in the hyperventilation response. Prolonged exercise‐induced hyperthermia (increased core temperature) is the primary stimulus increasing ventilation (${\dot{V}}_{E}$), in part as a result of sympathoadrenally mediated elevations in breathing frequency (*f*_b_). Dehydration alone (reduced body fluids), reductions in arterial partial pressure of CO_2_ ($P_{\mathrm{aC}O_{2}}$) and increases in arterial partial pressure of O_2_ ($P_{aO_{2}}$) and arterial pH (pH_a_) do not seem to affect ${\dot{V}}_{E}$. The hyperventilation is accompanied by compensatory adjustments in pulmonary gas exchange and peripheral tissues CO_2_ excretion and O_2_ extraction such that CO_2_ output (${\dot{V}}_{CO_{2}}$) is elevated and pulmonary O_2_ uptake (${\dot{V}}_{O_{2}}$) is maintained, despite the reduced pulmonary and systemic circulations. Arrows reflect the direction and magnitude of the effects. Abbreviations and symbols: (+), stimulatory effect on ${\dot{V}}_{E}$; (∅), no effect on ${\dot{V}}_{E}$; [A], plasma adrenaline; $F_{\mathrm{EC}O_{2}}$, expiratory carbon dioxide fraction; $F_{EO_{2}}$, expiratory oxygen fraction; $F_{IO_{2}}$, inspiratory oxygen fraction (0.2093); $F_{\mathrm{IC}O_{2}}$, inspiratory carbon dioxide fraction (0.0003); [NA], plasma noradrenaline. The photograph is from an endurance‐trained participant in study 1. The image of human lungs and heart was purchased from 123RF.com

Through retrospective analysis of a series of human‐based experiments, we demonstrated that: (1) hyperventilation is intimately related to elevations in core temperature and circulating catecholamines during endurance exercise, but not to changes in hydration status; (2) sympathoadrenal discharge is likely to contribute to hyperthermic hyperventilation during prolonged intense exercise in the heat; and (3) compensatory adjustments in pulmonary gas exchange occur during exercise in progressively dehydrated and hyperthermic humans, such that blood oxygenation and acid–base balance are preserved, despite blunting in pulmonary and systemic circulations. These findings illustrate the capacity of the respiratory system of endurance‐trained athletes to cope with the stress of reduced pulmonary circulation by augmenting respiratory rate and ventilation during prolonged intense exercise (Figure 9). This is likely to occur, at least in part, via sensing of central thermal stimuli and a catecholaminergically driven increase in central respiratory output.

## AUTHOR CONTRIBUTIONS

Studies 1–3 were performed at the then Human Physiology Department, August Krogh Institute, University of Copenhagen, Denmark, and the Department of Kinesiology and Health Education, University of Texas at Austin, Austin, TX, USA, as part of José González‐Alonso's postdoctoral and doctoral work at those universities, respectively. Study 4 formed part of Ricardo Mora‐Rodríguez's Master's thesis at the Department of Kinesiology and Health Education, University of Texas at Austin. José González‐Alonso drew the figures, analysed the data and wrote the first draft of the manuscript. Ricardo Mora‐Rodríguez analysed the data from study 4, and Pascale Kippelen calculated the alveolar ventilation data from study 1. José A. L. Calbet provided clinical expertise, contributed to the data collection and calculated the level of diffusion/perfusion limitation in study 1 according to the model of Piiper and Scheid (1981). All authors contributed to writing of subsequent drafts, approved the final version of the manuscript and agree to be accountable for all aspects of the work in ensuring that questions related to the accuracy or integrity of any part of the work are appropriately investigated and resolved. All persons designated as authors qualify for authorship, and all those who qualify for authorship are listed.

## CONFLICT OF INTEREST

None declared.

## Supporting information

Statistical Summary Document

## Data Availability

The data that support the findings of this study are available from the corresponding author upon reasonable request.
