# Supplementary material for: Pulmonary ventilation and gas exchange during prolonged exercise in humans: Influence of dehydration, hyperthermia and sympathoadrenal activity
Source: Exp Physiol. 2023 Jan 9;108(2):188–206. doi: 10.1113/EP090909 (PMC10103888; doi:10.1113/EP090909)
Supplement: Supplementary file 1 — Statistical Summary Document [file EPH-108-188-s001.pdf]

Experimental Physiology

Statistical Summary Document

Manuscript Title: Pulmonary ventilation and gas exchange during prolonged exercise in humans: influence of dehydration, hyperthermia and sympathoadrenal activity

Authors: José González-Alonso, Ricardo Mora-Rodríguez and Pascale Kippelen

Underlying hypotheses: We hypothesised that (1) compensatory adjustments in pulmonary gas exchange would occur during prolonged intense exercise in the heat, such that arterial blood gases and acid-base balance disturbances are minimised, (2) hyperthermia, but not dehydration, independently would increase ventilation during prolonged intense exercise, and (3) adrenaline infusion during prolonged exercise in the heat would significantly increase ventilation

Abbreviations

ABE, actual base excess; **$a\text{-}\bar{v}O_2$ diff,** arterial mixed-venous oxygen content difference; **C_aO_2**, arterial oxygen content; **$C\bar{v}O_2$**, mixed-venous oxygen content; **$C_{fv}O_2$**, femoral venous oxygen content; **$C\bar{v}CO_2$**, mixed-venous carbon dioxide content; **$C_{fv}CO_2$**, femoral venous carbon dioxide content; ***f_b***, breathing frequency; **$[Hb]_a$**, arterial blood haemoglobin concentration; **$[NA]_a$**, arterial noradrenaline concentration; **$[A]_a$**, arterial adrenaline concentration; **P_aO_2**, arterial partial pressure of oxygen; **P_aCO_2**, arterial partial pressure of carbon dioxide; **$P\bar{v}CO_2$**, mixed-venous partial pressure of carbon dioxide; **$\dot{Q}$**, cardiac output; **S_aO_2**, arterial oxygen saturation; **T_c**, core (oesophageal) temperature; **T_{sk}**, mean skin temperature; **V_D**, anatomical dead space; **$\dot{V}_E$**, pulmonary ventilation; **$\dot{V}_A$**, alveolar ventilation; **$\dot{V}_A/\dot{Q}$**, ventilation-perfusion ratio; **V_T**, tidal volume; **$\dot{V}O_2$**, oxygen consumption; **$\dot{V}CO_2$**, carbon dioxide output; **$\bar{v}\text{-}aCO_2$ diff**, mixed-venous arterial CO_2 content difference.

Definitions of 'n'

Question 1: n = number of ventilatory and systemic blood flow responses across time, hydration conditions and total comparisons
Question 2: n = number of blood gases and acid-base balance responses across time, hydration conditions and total comparisons
Question 3: n = number of blood contents, $a\text{-}\bar{v}$ differences, $\dot{V}O_2$ and $\dot{V}CO_2$ across time, hydration conditions and total comparisons
Question 4: n = number of systemic and leg blood gas contents, $\dot{V}CO_2$ and $\dot{V}O_2$ across time, hydration conditions and total comparisons
Question 5: n = number of data points compared in the regression analysis
Question 6: n = number of participants per comparison
Question 7: n = number of ventilatory responses across time, infusion type (adrenaline vs. saline) and total comparisons
Question 8: n = number of data points compared in the regression analysis

Question 9: n = number of temperature responses across time, hydration conditions and total comparisons
Question 10: n = number of blood volume and osmolality responses across time, hydration conditions and total comparisons
Question 11: n = number of expiratory and mixed venous gases responses across time, hydration conditions and total comparisons
Question 12: n = number of catecholamine responses across time, hydration conditions and total comparisons
Question 13: n = number of data points in the regression analysis
Question 14: n = number of participants per comparison
Question 15: n = number of participants per comparison
Question 16: n = number of participants per comparison
Question 17: n = number of participants per comparison
Question 18: n = number of participants per comparison
Question 19: n = number of participants per comparison
Question 20: n = number of participants per comparison
Question 21: n = number of participants per comparison
Question 22: n = number of participants per comparison
Question 23: n = number of participants per comparison
Question 24: n = number of participants per comparison
Question 25: n = number of participants per comparison
Question 26: n = number of participants per comparison
Question 27: n = number of participants per comparison
Question 28: n = number of participants per comparison
Question 29: n = number of temperature responses across time, infusion type (adrenaline vs. saline) and total comparisons
Question 30: n = number of $\dot{V}O_2$ and $\dot{V}CO_2$ responses across time, infusion type (adrenaline vs. saline) and total comparisons
Question 31: n = number of $P_{ET}CO_2$ responses across time, infusion type (adrenaline vs. saline) and total comparisons
Question 32: n = number of catecholamine responses across time, infusion type (adrenaline vs. saline) and total comparisons

Statistical summary table

Experimental question #	Finding/ conclusion	Experimental variable & units	Statistical test	Mean value	SD	n	Data comparisons	Main & interaction effects P**	Post-hoc results P**	Figure/ table comments
1. What are the ventilatory and systemic blood flow responses to prolonged exercise with progressive dehydration and hyperthermia and maintained euhydration control?	V_E, f_b, V_T, VA, Q and VA/Q exhibited significant differences overtime in the dehydration and hyperthermia condition (DH) and in very few cases in the euhydration control condition.<br><br>There were significant interactions between time and hydration conditions in all variables (Time x Hydration	V_E, l/min	Two-way ANOVA (time x condition) with repeated measures with Bonferroni post-hoc analysis	n/a	n/a	5	Time	**0.0204**	**Time vs. 20 min – DH (*)** 60, 90, 120 & 134 min: **0.0113; 0.0072; 0.0473; 0.0060**<br><br>**Time vs 20 min – Control (*)** 90, 120, 134 min: **0.0008; 0.0114; 0.0033**<br><br>**DH vs control (†)** 134 min: **0.0042**	Figure 1<br><br>N = 7 participants , except for VA and VA/Q N = 6 participants<br><br>Overtime differences were compared to 20 min of exercise.
						2	Hydration	**<0.0001**		
						10	Time x Hydration	**0.0030**		
		f_b, breaths/min				5	Time	**0.0001**	**Time vs 20 min – DH (*)** 90, 120 & 134 min: **0.0049; 0.0569; 0.0025**<br><br>**Time vs 20 min – Control (*)** 90 min: **0.0445**<br><br>**DH vs control (†)** 134 min: **0.0042**	
						2	Hydration	0.0874		
						10	Time x Hydration	**0.0035**		
		V_T, l				5	Time	0.8225	**Time vs 20 min – DH (*)** NS	
						2	Hydration	0.0599		
						10	Time x Hydration	0.1334		

	interactions),<br>except V_T.								**Time vs 20 min – Control (*)**<br>NS<br><br>**DH vs control (†)**<br>NS	
		V_A, l/min				5	Time	**<0.0001**	**Time vs 20 min – DH (*)** 60, 90, 120 & 134 min: **0.0052; 0.0089; 0.0163; 0.0045**<br><br>**Time vs 20 min – Control (*)**<br>60, 90, 120, 134 min: **0.0052; 0.0091; 0.0444; 0.0108**<br><br>**DH vs control (†)**<br>120, 134 min: **0.0192; 0.0016**	
						2	Hydration	**0.0009**		
						10	Time x Hydration	**<0.0001**		
		Q, l/min				5	Time	**>0.0001**	**Time vs 20 min – DH (*)** 120 & 134 min: **0.0143; 0.0038**<br><br>**Time vs 20 min – Control (*)**<br>NS<br><br>**DH vs control (†)**<br>90, 120 & 134 min: **0.0486; 0.0009; 0.0010**	
						2	Hydration	**0.0086**		
						10	Time x Hydration	**>0.0001**		
		VA/Q ratio				5	Time	**>0.0001**	**Time vs 20 min – DH (*)**	
						2	Hydration	**0.0007**		

						10	Time x Hydration	**>0.0001**	90, 120 & 134 min:<br>**0.0176; 0.028; 0.0002**<br><br>**Time vs 20 min – Control (*)**<br>NS<br><br>**DH vs control (†)**<br>90, 120 & 134 min:<br>**0.0302; 0.0045; >0.0001**	
2. What are the blood gases and acid-base balance responses to prolonged exercise with progressive dehydration and hyperthermia and maintained euhydration control?	PaO_2, $PaCO_2$, $[Hb]_a$, pH_a and $[HCO_3^-]_a$ exhibited significant differences overtime in the dehydration and hyperthermia condition (DH) and in very few cases in the euhydration control condition. Yet, SaO_2 and ABE_a were unchanged.	PaO_2, mmHg	Two-way ANOVA (time x condition) with repeated measures with Bonferroni post-hoc analysis	n/a	n/a	5	Time	**0.0154**	**Time vs 20 min – DH (*)**120 & 134 min:<br>**0.0086; 0.0074**<br><br>**Time vs 20 min – Control (*)**<br>NS<br><br>**DH vs control (†)**<br>120 & 134 min:<br>**0.0128; 0.0024**	Figure 2<br><br>N = 7 participants<br><br>Overtime differences were compared to 20 min of exercise.
						2	Hydration	**0.0046**		
						10	Time x Hydration	**0.0064**		
		$PaCO_2$, mmHg				5	Time	**<0.0001**	**Time vs 20 min – DH (*)**120 & 134 min:<br>**0.0017; 0.0004**<br><br>**Time vs 20 min – Control (*)**<br>NS<br><br>**DH vs control (†)**<br>60, 90, 120 & 134 min:<br>**0.0384; 0.0092; 0.01022; 0.0057**	
						2	Hydration	**<0.0031**		
						10	Time x Hydration	**0.0270**		
		SaO_2, %				5	Time	0.1490	**Time vs 20 min – DH**<br>NS	
						2	Hydration	**0.0228**		

	There were significant interactions between time and hydration conditions in most variables (Time x Hydration interactions), except SaO_2, pH_a and ABE_a					10	Time x Hydration	0.4366	**Time vs 20 min – Control (*)**<br>NS<br><br>**DH vs control (†)**<br>NS	
		$[Hb]_a$, g/l				5	Time	**0.0474**	**Time vs 20 min – DH (*)** NS<br><br>**Time vs 20 min – Control (*)** NS<br><br>**DH vs control (†)**<br>60, 90, 120 & 134 min: **0.0318; 0.0125; 0.0048; 0.0049**	
						2	Hydration	**0.0129**		
						10	Time x Hydration	**0.0007**		
		pH_a				5	Time	**<0.0001**	**Time vs 20 min – DH (*)** 134 min: **0.0076**<br><br>**Time vs 20 min – Control (*)** NS<br><br>**DH vs control (†)**<br>134 min: **0.0353**	
						2	Hydration	**0.0061**		
						10	Time x Hydration	0.1664		
		$[HCO_3^-]_a$, mmol/l				5	Time	**0.0034**	**Time vs 20 min – DH (*)** NS<br><br>**Time vs 20 min – Control (*)** NS<br><br>**DH vs control (†)**<br>134 min: **0.0029**	
						2	Hydration	**0.0220**		
						10	Time x Hydration	**0.0064**		
		ABE_a, mmol/l				5	Time	0.6544	**Time vs 20 min – DH (*)** NS	
						2	Hydration	0.8666		
						10	Time x Hydration	0.2477		

									**Time vs 20 min – Control (*)** NS<br><br>**DH vs control (†)** NS	
3. What are the blood gas contents, a-v difference, VO_2 and VCO_2 responses to prolonged exercise with progressive dehydration and hyperthermia and maintained euhydration control?	$CaCO_2$, $C\bar{v}CO_2$, $\bar{v}$-aCO_2 diff, VCO_2, CaO_2, $C\bar{v}O_2$, a-$\bar{v}O_2$ diff and VO_2 exhibited significant differences overtime in the dehydration and hyperthermia condition (DH) and in very few cases in the euhydration control condition.<br><br>There were significant interactions between time and hydration conditions in all variables (Time x	$CaCO_2$, ml/l	Two-way ANOVA (time x condition) with repeated measures with Bonferroni post-hoc analysis	n/a	n/a	5	Time	**0.0141**	**Time vs 20 min – DH (*)** 134 min **0.0485**<br><br>**Time vs 20 min – Control (*)** NS<br><br>**DH vs control (†)** 120 & 134 min: **0.0021; 0.0035**	Figure 3<br><br>N = 7 participants<br><br>Overtime differences were compared to 20 min of exercise.
						2	Hydration	**0.0163**		
						10	Time x Hydration	**0.0062**		
		$C\bar{v}CO_2$, ml/l				5	Time	**0.0142**	**Time vs 20 min – DH (*)** NS<br><br>**Time vs 20 min – Control (*)** NS<br><br>**DH vs control (†)** NS	
						2	Hydration	0.6321		
						10	Time x Hydration	0.1518		
		$\bar{v}$-aCO_2 diff, ml/l				5	Time	**0.0006**	**Time vs 20 min – DH (*)** 120 & 134 min **0.0187; 0.0017**<br><br>**Time vs 20 min – Control (*)** NS<br><br>**DH vs control (†)** 90, 120 & 134 min: **0.0440; 0.0025; <0.0001**	
						2	Hydration	**0.0032**		
						10	Time x Hydration	**<0.0001**		
		VCO_2, l/min				5	Time	**0.0006**	**Time vs 20 min – DH (*)** NS	
						2	Hydration	**0.0365**		
						10	Time x Hydration	**0.0058**		

	Hydration interactions), except $C\bar{v}CO_2$.								**Time vs 20 min – Control (*)** NS<br><br>**DH vs control (†)**<br>134 min: **0.0058**	
		CaO_2, ml/l				5	Time	**0.0001**	**Time vs 20 min – DH (*)** 120 & 134 min: **0.0144; 0.0098**<br><br>**Time vs 20 min – Control (*)** NS<br><br>**DH vs control (†)**<br>134 min: **0.0398**	
						2	Hydration	**0.0131**		
						10	Time x Hydration	**<0.0001**		
		$C\bar{v}O_2$, ml/l				5	Time	**0.0001**	**Time vs 20 min – DH (*)** 120 & 134 min: **0.0309; 0.0096**<br><br>**Time vs 20 min – Control (*)** NS<br><br>**DH vs control (†)**<br>120 & 134 min:<br>**0.0008; 0.0024**	
						2	Hydration	**0.0331**		
						10	Time x Hydration	**<0.0001**		
		a-$\bar{v}O_2$ diff, ml/l				5	Time	**0.0241**	**Time vs 20 min – DH (*)** 90, 120 & 134 min: **0.0257; 0.0013; 0.0005**<br><br>**Time vs 20 min – Control (*)** NS<br><br>**DH vs control (†)**<br>120 & 134 min:<br>**0.0013; 0.0017**	
						2	Hydration	**0.0149**		
						10	Time x Hydration	**<0.0001**		

		VO_2, l/min				5	Time	**<0.0001**	**Time vs 20 min – DH (*)** 120 & 134 min: **0.0168; 0.0060**<br><br>**Time vs 20 min – Control (*)** 90, 120 & 134 min: **0.0011; 0.0014; 0.0007**<br><br>**DH vs control (†)** NS	
						2	Hydration	0.4692		
						10	Time x Hydration	**0.0028**		
4. What are the systemic and leg blood gas contents, VCO_2 and VO_2 responses to prolonged exercise with progressive dehydration and hyperthermia and maintained euhydration control?	$C\bar{v}CO_2$, $CfvCO_2$, $C\bar{v}O_2$, $CfvO_2$, VCO_2 and VO_2 exhibited significant differences overtime in the dehydration and hyperthermia condition (DH) and in very few cases in the euhydration control condition.<br><br>There were significant interactions	$C\bar{v}CO_2$, ml/l	Two-way ANOVA (time x condition) with repeated measures with Bonferroni post-hoc analysis	n/a	n/a	5	Time	0.0622	**Time vs 20 min – DH (*)** NS<br><br>**Time vs 20 min – Control (*)** NS<br><br>**DH vs control (†)** NS	Figure 4<br><br>N = 7 participants<br><br>Overtime differences were compared to 20 min of exercise.
						2	Hydration	0.6321		
						10	Time x Hydration	0.1518		
		$CfvCO_2$, ml/l				5	Time	**0.0022**	**Time vs 20 min – DH (*)** NS<br><br>**Time vs 20 min – Control (*)** NS<br><br>**DH vs control (†)** NS	
						2	Hydration	0.6372		
						10	Time x Hydration	0.1574		
		$C\bar{v}O_2$, ml/l				5	Time	**<0.0001**	**Time vs 20 min – DH (*)**120 & 134 min: **0.0166; 0.0049**<br><br>**Time vs 20 min – Control (*)** NS<br><br>**DH vs control (†)**	
						2	Hydration	0.9733		
						10	Time x Hydration	**<0.0001**		

	between time and hydration conditions in all variables (Time x Hydration interactions). conditions in many variables, except $C\bar{v}CO_2$, $CfvCO_2$ and $CfvO_2$								120 & 134 min: **0.0301; 0.0227**	
		$CfvO_2$, ml/l				5	Time	**<0.0001**	**Time vs 20 min – DH (*)** NS<br><br>**Time vs 20 min – Control (*)** NS<br><br>**DH vs control (†)** NS	
						2	Hydration	0.5383		
						10	Time x Hydration	0.7370		
		Systemic VCO_2, l/min				5	Time	**0.0006**	**Time vs 20 min – DH (*)** NS<br><br>**Time vs 20 min – Control (*)** NS<br><br>**DH vs control (†)** 134 min: **0.0058**	
						2	Hydration	**0.0365**		
						10	Time x Hydration	**0.0058**		
		Legs VCO_2, l/min				5	Time	0.8053	**Time vs 20 min – DH (*)** NS<br><br>**Time vs 20 min – Control (*)** NS<br><br>**DH vs control (†)** NS	
						2	Hydration	0.7708		
						10	Time x Hydration	0.3314		
		Systemic VO_2, l/min				5	Time	**<0.0001**	**Time vs 20 min – DH (*)** 120 & 134 min: **0.0168; 0.0060**<br><br>**Time vs 20 min – Control (*)** 90, 120 & 134 min: **0.0011; 0.0014; 0.0007**<br><br>**DH vs control (†)**	
						2	Hydration	0.4692		
						10	Time x Hydration	**0.0028**		

									NS	
		Legs VO_2, l/min				5	Time	**0.0251**	**Time vs 20 min – DH (*)** NS<br><br>**Time vs 20 min – Control (*)** NS<br><br>**DH vs control (†)** NS	
						2	Hydration	0.8373		
						10	Time x Hydration	0.1576		
5.What are the relationships between VE and f_b vs. core temperature and arterial catecholamines during prolonged exercise with progressive dehydration and hyperthermia (DH) and maintained euhydration control (control)?	There were significant direct relationships between increases in V_E and fb with the increases in core temperature and combined catecholamines	V_E vs. T_c, l/min & °C	Regression analysis	n/a	n/a	5	DH	R^2 =0.994	P = **0.0002**	Figure 5<br><br>DH vs. control, 22 participants
						5	Control	R^2 =0.818	P = **0.0348**	
		f_b vs. T_c, breaths/min & °C				5	DH	R^2 =0.982	P = **0.0010**	
						5	Control	R^2 =0.854	P = **0.0243**	
		VE vs. arterial catecholamines, l/min & mmol/l				5	DH	R^2 =0.972	P = **0.001**	
						5	Control	R^2 =0.908	P = **0.012**	
		f_b vs. arterial catecholamines, breaths/min & mmol/l				5	DH	R^2 =0.973	P = **0.001**	
						5	Control	R^2 =0.898	P = **0.014**	
6. What are the ventilatory effects of combined (DH) and separate dehydration (D) and/or	DH and H induced significant increases in V_E and fb, but not VT. In contrast, D did not alter	ΔV_E, l/min	One-way (condition) ANOVA with repeated measures with Bonferroni post-hoc analysis	5.0	2.2	22	DH vs. control	**0.0001**	n/a	Figure 6<br><br>DH vs. control, 22 participants H vs. control, 7 participants
				5.7	2.3	7	H vs. control	**0.001**		
				0.8	3.5	8	D vs. control	0.361		
		Δ f_b, breaths/min		4	3	22	DH vs. control	**0.0001**		
				3	2	7	H vs. control	**0.007**		
				2.4	3	8	D vs. control	**0.005**		
		ΔV_T, ml		90	150	22	DH vs. control	**0.009**		
				10	70	7	H vs. control	0.793		

hyperthermia (H)?	these responses.			175	200	8	D vs. control	**0.001**		D vs. control, 8 participants
7. What are the ventilatory responses to adrenaline (A) and saline (S) infusion during prolonged exercise with progressive dehydration and hyperthermia?	VE, f_b, V_T, exhibited significant differences overtime in the adrenaline infusion (A and some in the control saline (S) control condition.<br><br>Time (min) x condition (A vs. S control) interactions. There was a significant interaction between time and conditions in VE, but not in f_b and V_T.	V_E, l/min	Two-way (time x condition) ANOVA with repeated measures with Bonferroni post-hoc analysis	n/a	n/a	8	Time	**<0.0001**	**Time vs 10 min – A infusion (*)**<br>45, 85 & 120 min: **0.0016; 0.0269; 0.0252**<br><br>**Time vs 10 min – S infusion (*)** 45, 85 & 120 min: **0.0122; 0.0674; 0.0282**<br><br>**A vs S control (†)**<br>45, 85 & 120 min: **0.0063; 0.0077; 0.0672**	Figure 7<br><br>N= 7 participants
						2	Infusion	**0.0140**		
						16	Time x Infusion	**0.0016**		
		f_b, breaths/min				8	Time	**<0.0001**	**Time vs 10 min – A infusion (*)**<br>45, 85 & 120 min: **0.0196; 0.0201; 0.0130**<br><br>**Time vs 10 min – S infusion (*)** 45, 85 & 120 min: **0.0123; 0.0029; 0.0068**<br><br>**A vs S control (†)**<br>NS	
						2	Infusion	0.1214		
						16	Time x Infusion	**0.1546**		
		V_T, ml				8	Time	0.1471	**Time vs 10 min – A infusion (*)**<br>NS	
						2	Infusion	0.2891		
							Time x Infusion	0.0733		

									**Time vs 10 min – S infusion (*)** NS<br><br>**A vs S control (†)** NS	
8. What are the relationships between VE and f_b vs. core temperature and arterial catecholamines with adrenaline (A) and saline (S) infusion during prolonged exercise with dehydration & hyperthermia?	There were significant direct relationships between increases in V_E and fb with the increases in core temperature and combined catecholamines	V_E vs. T_c, l/min & °C	Regression analysis	n/a	n/a	4	Adrenaline	R^2 =0.990	P = **0.004**	Figure 8<br><br>N = 7 participants
						4	Saline	R^2 =0.996	P = **0.001**	
		f_b vs. Tc, breaths/min & °C				4	Adrenaline	R^2 =0.983	P = **0.008**	
						4	Saline	R^2 =0.970	P = **0.014**	
		V_E vs. arterial catecholamines, l/min & mmol/l				4	Adrenaline	R^2 =0.986	P = **0.006**	
						4	Saline	R^2 =0.932	P = **0.034**	
		f_b vs. arterial catecholamines, breaths/min & mmol/l				4	Adrenaline	R^2 =0.950	P = **0.024**	
						4	Saline	R^2 =0.969	P = **0.015**	
9. What are the effects of dehydration on core and skin temperature during prolonged exercise compared to euhydration control?	Dehydration significantly increase T_c after 90 min of exercise while T_{sk} remained unchanged.	T_c, °C	Two-way (time x condition) ANOVA with repeated measures with Bonferroni post-hoc analysis	n/a	n/a	5	Time	**<0.0001**	**Time vs 20 min – DH (*)** 90, 120 & 134 min; 0.0531; **0.0047; 0.0005**<br><br>**Time vs 20 min – Control (*)** NS<br><br>**DH vs control (†)** 90, 120 & 134 min: **0.0084; 0.0005; 0.0002**	Table 2<br><br>N = 7 participants<br><br>Overtime differences were compared to 20 min of exercise.
						2	Hydration	**0.0021**		
						10	Time x Hydration	**<0.0001**		
		T_{sk}, °C				5	Time	**0.0003**	**Time vs 20 min – DH (*)** NS	

						2	Hydration	**0.0365**	**Time vs 20 min – Control (*)** NS<br><br>**DH vs control (†)** NS	
						10	Time x Hydration	0.8446		
10. What are the effects of dehydration on blood volume and osmolality during prolonged exercise compared to euhydration control?	BV & osmolality exhibited significant differences overtime in the dehydration and hyperthermia condition (DH) and some in the control condition.<br><br>Time (min) x condition (DH vs. control) interactions. There were significant interactions between time and conditions in both variables	Blood volume, l	Two-way (time x condition) ANOVA with repeated measures with Bonferroni post-hoc analysis	n/a	n/a	5	Time	**0.0065**	**Time vs 20 min – DH (*)**134 min; **0.0487**<br><br>**Time vs 20 min – Control (*)** NS<br><br>**DH vs control (†)** 60, 90, 120 & 134 min: **0.0306; 0.0018; 0.0006; 0.0004**	Table 2<br><br>N = 7 participants<br><br>Overtime differences were compared to 20 min of exercise.
						2	Hydration	**0.0027**		
						10	Time x Hydration	**0.0004**		
		Osmolality, mOsm/kg				5	Time	0.5785	**Time vs 20 min – DH (*)** 60, 90, 120 & 134 min: **0.0075; 0.0168; 0.0015; 0.0035**<br><br>**Time vs 20 min – Control (*)** 134 min: **0.0059**<br><br>**DH vs control (†)** 60, 90, 120 & 134 min: **0.0043; 0.0002; 0.0002; 0.0001**	
						2	Hydration	**0.0003**		
						10	Time x Hydration	**<0.0001**		

11. What are the effects of dehydration and hyperthermia (DH) on end-tidal gases during prolonged exercise compared to euhydration control?	$P_{ET}CO_2$, $P\bar{v}O_2$ & $P_{ET}O_2$ exhibited significant differences overtime in the dehydration and hyperthermia condition (DH) and some in the control condition.<br><br>Time (min) x condition (DH vs. control) interactions. There were significant interactions between time and conditions in $P_{ET}CO_2$ and $P_{ET}O_2$ but not in $P\bar{v}O_2$.	$P_{ET}CO_2$, mmHg	Two-way (time x condition) ANOVA with repeated measures with Bonferroni post-hoc analysis	n/a	n/a	5	Time	**0.0013**	**Time vs 20 min – DH (*)** 60, 90, 120 & 134 min: **0.0300; 0.0003; 0.0067; 0.0253**<br><br>**Time vs 20 min – Control (*)**60, 90, 120 & 134 min: **0.0299; 0.0096; 0.0043; 0.0040**<br><br>**DH vs control (†)** 120 & 134 min: **0.0043; 0.0040**	Table 2<br><br>N = 7 participants<br><br>Overtime differences were compared to 20 min of exercise.
						2	Hydration	**0.0320**		
						10	Time x Hydration	**0.0054**		
		$P\bar{v}O_2$, mmHg				5	Time	**0.0029**	**Time vs 20 min – DH (*)** 120 & 134 min: **0.0114; 0.0315**<br><br>**Time vs 20 min – Control (*)** 120 & 134 min: **0.0015; 0.0484**<br><br>**DH vs control (†)** NS	
						2	Hydration	**0.0318**		
						10	Time x Hydration	0.2565		
		$P_{ET}O_2$, mmHg				5	Time	**0.0031**	**Time vs 20 min – DH (*)** 60, 90, 120 & 134 min: **0.0028; 0.0043; 0.0156; 0.0500**<br><br>**Time vs 20 min – Control (*)** NS<br><br>**DH vs control (†)** 134 min: **0.0311**	
						2	Hydration	0.0618		
						10	Time x Hydration	**0.0448**		
12. What are the effects of	Plasma catecholamin	[NA], mmol/l	Two-way (time x condition)	n/a	n/a	5	Time	**<0.0001**	**Time vs 20 min – DH (*)** 60, 90, 120 & 134	Table 2

dehydration on circulating catecholamines during prolonged exercise compared to euhydration control?	es exhibited significant differences overtime in the dehydration and hyperthermia condition (DH) and some in the control condition<br><br>Time (min) x condition (DH vs. control) interactions. There were significant interactions between time and conditions in [NA] but not [A].		ANOVA with repeated measures with Bonferroni post-hoc analysis			2	Hydration	**0.0076**	min: **0.0075; 0.0011; 0.0001; 0.0007**<br><br>**Time vs 20 min – Control (*)** NS<br><br>**DH vs control (†)** 60,90, 120 134 min: **0.0247; 0.0417; 0.0049; 0.0012**	N = 7 participants<br><br>Overtime differences were compared to 20 min of exercise.
						10	Time x Hydration	**<0.0001**		
		[A], mmol/l				5	Time	**<0.0001**	**Time vs 20 min – DH (*)** 134 min: **0.0466**	
						2	Hydration	**0.0040**	**Time vs 20 min – Control (*)** NS<br><br>**DH vs control (†)** 90, 120 134 min: **0.0177; 0.0445; 0.0313**	
						10	Time x Hydration	0.1401		
13. What are the effects of combined dehydration and hyperthermia (DH) on body temperatures	DH induced significant increases in T_c with smaller increases in T_{sk} compared to control.	T_c control, °C	One-way ANOVA (condition) with repeated measures with Bonferroni post-hoc analysis	38.2	0.4	22	DH vs control	**<0.0001**	n/a	Table 3<br><br>DH vs. control, 22 participants
		T_c DH, °C		39.1	0.4	22				
		T_{sk} control, °C		34.2	0.6	22		**0.0021**		
		T_{sk} DH, °C		34.7	0.9	22				

during prolonged exercise compared to euhydration control?										
14. What are the effects of combined dehydration and hyperthermia (DH) on % body mass loss, haemoglobin and blood osmolality during prolonged exercise compared to euhydration control?	DH induced significant increases in %BM loss, [Hb] and osmolality compared to control.	%BM loss control	One-way ANOVA (condition) with repeated measures with Bonferroni post-hoc analysis	-0.5	0.3	22	DH vs control	**<0.0001**	n/a	Table 3<br><br>DH vs. control, 22 participants
		%BM loss DH		-4.6	0.5	22				
		[Hb] control, g/l		159	8	22		**<0.0001**		
		[Hb] DH, g/l		168	10	22				
		Osmolality control, mOsm/kg		278	4	22		**<0.0001**		
		Osmolality DH, mOsm/kg		298	4	22				
15. What are the effects of combined dehydration and hyperthermia (DH) on metabolism during prolonged exercise compared to	DH did not significantly alter VO_2 or VCO_2 compared to control.	VO_2 control, l/min	One-way ANOVA (condition) with repeated measures with Bonferroni post-hoc analysis	3.09	0.25	22	DH vs control	0.1925	n/a	Table 3<br><br>DH vs. control, 22 participants
		VO_2 DH, l/min		3.10	0.26	22				
		VCO_2 control, l/min		2.94	0.26	22		0.0895		
		VCO_2 DH, l/min		2.97	0.26	22				

euhydration control?										
16. What are the effects of combined dehydration and hyperthermia (DH) on the ventilatory responses during prolonged exercise compared to euhydration control?	DH significantly increased V_E, f_b and V_T, and tended to increase $P_{ET}CO_2$ compared to control.	V_E control, l/min	One-way ANOVA (condition) with repeated measures with Bonferroni post-hoc analysis	68.6	6.2	22	DH vs control	**<0.0001**	n/a	Table 3<br><br>DH vs. control, 22 participants
		V_E DH, l/min		73.5	6.9	22				
		f_b control, breaths/min		34	6	22		**<0.0001**		
		f_b DH, breaths/min		38	7	22				
		V_T control, l		2.05	0.24	22		**0.0086**		
		V_T DH, l		1.96	0.26	22				
		$P_{ET}CO_2$ control, mmHg		37	4	22		0.0605		
		$P_{ET}CO_2$ DH, mmHg		35	4	22				
17. What are the effects of isolated hyperthermia (H) on body temperature responses during prolonged exercise compared to euhydration control?	As per design, H induced significant increases in T_c with smaller increases in T_{sk} compared to control.	T_c control, °C	One-way ANOVA (condition) with repeated measures with Bonferroni post-hoc analysis	38.3	0.2	7	H vs control	**0.0001**	n/a	Table 3<br><br>H vs. control, 7 participants
		T_c H, °C		39.2	0.3	7				
		T_{sk} control, °C		34.0	0.6	7		**0.0260**		
		T_{sk} H, °C		34.6	0.8	7				
18. What are the effects of isolated hyperthermia (H) on % body	As per design, H did not significantly change %BM	%BM loss control	One-way ANOVA (condition) with repeated measures with	-0.5	0.3	7	H vs control	0.0707	n/a	Table 3<br><br>H vs. control, 7 participants
		%BM loss H		-4.6	0.5	7				
		[Hb] control, g/l		159	8	7		**0.0488**		

mass loss, haemoglobin and blood osmolality responses during prolonged exercise compared to euhydration control?	loss or osmolality compared to control.	[Hb] H, g/l	Bonferroni post-hoc analysis	168	10	7				
		Osmolality control, mOsm/kg		278	4	7		0.2140	n	
		Osmolality H, mOsm/kg		298	4	7				
19. What are the effects of isolated hyperthermia (H) on metabolism during prolonged exercise compared to euhydration control?	H did not alter VO_2 but reduced VCO_2 compared to control.	VO_2 control, l/min	One-way ANOVA (condition) with repeated measures with Bonferroni post-hoc analysis	3.15	0.28	7	H vs control	0.3955	n/a	Table 3<br><br>DH vs. control, 22 participants
		VO_2 H, l/min		3.16	0.27	7				
		VCO_2 control, l/min		3.05	0.29	7		**0.0222**		
		VCO_2 H, l/min		2.93	0.21	7				
20. What are the effects of isolated hyperthermia (H) on the ventilatory responses during prolonged exercise compared to euhydration control?	DH significantly increased V_E, f_b and $P_{ET}CO_2$ while V_T remained unchanged compared to control.	V_E control, l/min	One-way ANOVA (condition) with repeated measures with Bonferroni post-hoc analysis	68.5	4.9	7	H vs control	**0.0007**	n/a	Table 3<br><br>DH vs. control, 22 participants
		V_E H, l/min		74.2	6.6	7				
		f_b control, breaths/min		35	6	7		**0.0066**		
		f_b H, breaths/min		38	6	7				
		V_T control, l		2.00	0.25	7		0.7933		
		V_T H, l		1.99	0.24	7				
		$P_{ET}CO_2$ control, mmHg		38	4	7		**0.0056**		
		$P_{ET}CO_2$ DH, mmHg		35	3	7				

21. What are the effects of isolated dehydration (D) on body temperature responses during prolonged exercise compared to euhydration control?	As per design, D did not increase T_c or T_{sk} compared to control.	T_c control, °C	One-way ANOVA (condition) with repeated measures with Bonferroni post-hoc analysis	38.1	0.4	8	D vs control	**0.0656**	n/a<br>n/a	Table 3<br><br>D vs. control, 8 participants
		T_c D, °C		38.2	0.3	8				
		T_{sk} control, °C		21.2	1.3	8		0.1266		
		T_{sk} D, °C		20.4	1.2	8				
22. What are the effects of isolated hyperthermia (D) on % body mass loss, haemoglobin and blood osmolality responses during prolonged exercise compared to euhydration control?	As per design, D reduced %BM loss and increased [Hb] and osmolality compared to control.	%BM loss control	One-way ANOVA (condition) with repeated measures with Bonferroni post-hoc analysis	-0.1	0.2	8	D vs control	**<0.0001**	n/a	Table 3<br><br>D vs. control, 8 participants
		%BM loss D		-4.2	0.3	8				
		[Hb] control, g/l		156	8	8		**<0.0001**		
		[Hb] D, g/l		164	7	8				
		Osmolality control, mOsm/kg		281	3	8		**<0.0001**		
		Osmolality D, mOsm/kg		296	5	8				
23. What are the effects of isolated dehydration (D) on metabolism during	D did not alter either VO_2 or VCO_2 compared to control.	VO_2 control, l/min	One-way ANOVA (condition) with repeated measures with Bonferroni	3.22	0.34	8	D vs control	0.2662	n/a	Table 3<br><br>D vs. control, 8 participants
		VO_2 D, l/min		3.20	0.34	8				
		VCO_2 control, l/min		3.04	0.34	8		0.2337		
		VCO_2 D, l/min		2.99	0.31	8				

prolonged exercise compared to euhydration control?			post-hoc analysis							
24. What are the effects of isolated hyperthermia (H) on the ventilatory responses during prolonged exercise compared to euhydration control?	D did not alter V_E, f_b or $P_{ET}CO_2$, but reduced V_T compared to control.	V_E control, l/min	One-way ANOVA (condition) with repeated measures with Bonferroni post-hoc analysis	67.5	5.8	8	D vs control	0.7194	n/a	Table 3<br><br>DH vs. control, 22 participants
		V_E DH, l/min		68.1	5.5	8				
		f_b control, breaths/min		30	4	8		0.0807		
		f_b DH, breaths/min		32	4	8				
		V_T control, l		2.31	0.47	8		**0.0442**		
		V_T DH, l		2.14	0.32	8				
		$P_{ET}CO_2$ control, mmHg		39	6	8		0.8994		
		$P_{ET}CO_2$ DH, mmHg		39	4	8				
25. What are the effects of isolated dehydration and BV restoration (D+BV restoration) on body temperature responses during prolonged exercise compared to euhydration control?	D+BV restoration did not increase T_c or T_{sk} compared to control.	T_c control, °C	One-way ANOVA (condition) with repeated measures with Bonferroni post-hoc analysis	38.0	0.4	8	D+BV restoration vs control	0.1705	n/a	Table 3<br><br>D+BV vs. control, 8 participants
		T_c D+BV restoration, °C		38.1	0.3	8				
		T_{sk} control, °C		20.9	1.4	8		0.9762		
		T_{sk} D+BV restoration, °C		20.9	0.9	8				

26. What are the effects of isolated dehydration and BV restoration (D+BV restoration) on % body mass loss, haemoglobin and blood osmolality responses during prolonged exercise compared to euhydration control?	As per design, D + BV restoration reduced %BM loss and [Hb] increased [Hb] while osmolality remained elevated compared to control.	%BM loss control	One-way ANOVA (condition) with repeated measures with Bonferroni post-hoc analysis	-0.3	0.2	8	D+BV restoration vs control	**<0.0001**	n/a	Table 3<br><br>D+BV vs. control, 8 participants
		%BM loss D+BV restoration		-4.2	0.4	8				
		[Hb] control, g/l		156	8	8		**0.0221**		
		[Hb] D+BV restoration, g/l		153	9	8				
		Osmolality control, mOsm/kg		281	3	8		**0.0001**		
		Osmolality D+BV restoration, mOsm/kg		296	5	8				
27. What are the effects of isolated dehydration and BV restoration (D+BV restoration) on metabolism during prolonged exercise compared to euhydration control?	D+BV restoration did not alter VO_2 but reduced VCO_2 compared to control.	VO_2 control, l/min	One-way ANOVA (condition) with repeated measures with Bonferroni post-hoc analysis	3.21	0.34	8	D+BV restoration vs control	0.7560	n/a	Table 3<br><br>D+BV vs. control, 8 participants
		VO_2 D+BV restoration, l/min		3.22	0.33	8				
		VCO_2 control, l/min		3.00	0.33	8		**0.0065**		
		VCO_2 D+BV restoration, l/min		2.95	0.31	8				

28. What are the effects of isolated dehydration and BV restoration (D+BV restoration) on the ventilatory responses during prolonged exercise compared to euhydration control?	D+BV restoration did not alter V_E or $P_{ET}CO_2$, but increased f_b and reduced V_T compared to control.	V_E control, l/min	One-way ANOVA (condition) with repeated measures with Bonferroni post-hoc analysis	65.5	6.0	8	D+BV restoration vs control	0.3689	n/a	Table 3<br><br>D+BV vs. control, 8 participants
		V_E D+BV restoration, l/min		66.6	4.9	8				
		f_b control, breaths/min		30	5	8		**0.0385**		
		f_b D+BV restoration, breaths/min		33	3	8				
		V_T control, l		2.25	0.46	8		**0.0133**		
		V_T D+BV restoration, l		2.08	0.37	8				
		$P_{ET}CO_2$ control, mmHg		39	4	8		0.8357		
		$P_{ET}CO_2$ D+BV restoration, mmHg		39	3	8				
29. What are the effects of adrenaline (A) infusion on body temperature responses during prolonged exercise compared to saline (S) infusion control?	T_c exhibited significant differences overtime in the A and S, but T_{sk} remained unchanged.<br><br>Time (min) x condition (A vs. S) interactions. There were significant interactions between time and	T_c, °C	Two-way ANOVA with repeated measures with Bonferroni post-hoc analysis	n/a	n/a	4	Time	**<0.0001**	**Time vs 10-30 min – DH (*)** 40-60, 85-90 & 120 min: **<0.0001; 0.0013; 0.0001**<br><br>**Time vs 10-30 min – Control (*)** 40-60, 85-90 & 120 min: **0.0026; 0.0001; 0.0002**<br><br>**A vs S control (†)** 40-60, 85-90 & 120 min: **0.0009; 0.0127; 0.0066**	Table 4<br><br>N = 7 participants<br><br>Overtime differences were compared to 10 min of exercise.
						2	Infusion	**0.0029**		
						8	Time x Infusion	**0.0007**		
		T_{sk}, °C				4	Time	**0.0014**	**Time vs 10-30 min – DH (*)** NS	
						2	Infusion	**0.0303**		

	conditions in T_c but not in T_{sk}.					8	Time x Infusion	0.8743	**Time vs 10-30 min – Control (*)** NS<br><br>**A vs S control (†)** NS	
30. What are the effects of adrenaline (A) infusion on VO_2 and VCO_2 responses during prolonged exercise compared to saline (S) infusion control?	VO_2 and VCO_2 exhibited significant differences overtime in the A and S.<br><br>Time (min) x condition (A vs. S) interactions. There were significant interactions between time and conditions in both VO_2 and VCO_2.	VO_2, l/min	Two-way ANOVA with repeated measures with Bonferroni post-hoc analysis	n/a	n/a	4	Time	**0.0003**	**Time vs 10-30 min – A (*)** 40-60, 85-90 & 120 min: **0.0154; 0.040; 0.0392**<br><br>**Time vs 10-30 min – Control (*)** 40-60, 85-90 & 120 min: **0.0180; 0.0450; 0.0499**<br><br>**A vs S control (†)** NS	Table 4<br><br>N = 7 participants<br><br>Overtime differences were compared to 10 min of exercise.
						2	Infusion	0.1194		
						8	Time x Infusion	0.1031		
		VCO_2, l/min				4	Time	**0.0036**	**Time vs 10-30 min – A (*)** 40-60, 85-90 & 120 min: **0.0154; 0.040; 0.0392**<br><br>**Time vs 10-30 min – Control (*)** NS<br><br>**A vs S control (†)** 40-60, 85-90 & 120 min: **<0.0001; 0.0105; 0.0196**	
						2	Infusion	**0.0222**		
						8	Time x Infusion	**0.0024**		
31. What are the effects of adrenaline (A) infusion on end-tidal PCO_2 responses during	$P_{ET}CO_2$ exhibited significant differences overtime in the A and S.	$P_{ET}CO_2$, mmHg	Two-way ANOVA with repeated measures with Bonferroni post-hoc analysis	n/a	n/a	4	Time	**0.0301**	**Time vs 10-30 min – A (*)** 40-60 & 85-90 min: **0.0011; 0.0044**<br><br>**Time vs 10-30 min – Control (*)** NS	Table 4<br><br>N = 7 participants<br><br>Overtime differences
						2	Infusion	0.4991		

prolonged exercise compared to saline (S) infusion control?	Time (min) x condition (A vs. S) interactions. There were significant interactions between time and conditions in $P_{ET}CO_2$..					8	Time x Infusion	**0.0288**	**A vs S control (†)** 40-60 min: **<0.0063**	were compared to 10 min of exercise.
32. What are the effects of adrenaline (A) infusion on circulating catecholamines responses during prolonged exercise compared to saline (S) infusion control?	Plasma catecholamines exhibited significant differences overtime in the A and to lesser extend in S. Time (min) x condition (A vs. S) interactions. There were significant interactions between time and conditions in [A] but not in [NA].	[NA], mmol/l	Two-way ANOVA with repeated measures with Bonferroni post-hoc analysis	n/a	n/a	4	Time	**0.0069**	**Time vs 10-30 min – A (*)** 85-90 & 120 min: **0.0390; 0.0422** **Time vs 10-30 min – Control (*)** NS **A vs S control (†)** NS	Table 4 N = 7 participants Overtime differences were compared to 10 min of exercise.
						2	Infusion	0.6384		
						8	Time x Infusion	0.4303		
		[A], mmol/l				4	Time	**<0.0001**	**Time vs 10-30 min – A (*)** 40-60, 85-90 & 120 min: **0.0081; 0.0120; 0.0373** **Time vs 10-30 min – Control (*)**120 min: **0.0222** **A vs S control (†)** 40-60; 85-90 & 120 min: **0.0013; 0.0020; 0.0130**	
						2	Infusion	**0.0025**		
						8	Time x Infusion	**<0.0001**		
